# Comparative compositional and functional analyses of *Bothrops moojeni* specimens reveal several individual variations

**DOI:** 10.1371/journal.pone.0222206

**Published:** 2019-09-12

**Authors:** Weslei da Silva Aguiar, Nathália da Costa Galizio, Caroline Serino-Silva, Sávio Stefanini Sant’Anna, Kathleen Fernandes Grego, Alexandre Keiji Tashima, Erika Sayuri Nishiduka, Karen de Morais-Zani, Anita Mitico Tanaka-Azevedo

**Affiliations:** 1 Laboratório de Herpetologia, Instituto Butantan, São Paulo, Brasil; 2 Interunidades em Biotecnologia, Universidade de São Paulo, Instituto de Pesquisas Tecnológicas, Instituto Butantan, São Paulo, Brasil; 3 Departamento de Bioquímica, Universidade Federal de São Paulo, São Paulo, Brasil; Universidade Federal do Rio de Janeiro, BRAZIL

## Abstract

Snake venoms are complex protein mixtures with different biological activities that can act in both their preys and human victims. Many of these proteins play a role in prey capture and in the digestive process of these animals. It is known that some snakes are resistant to the toxicity of their own venom by mechanisms not yet fully elucidated. However, it was observed in the Laboratory of Herpetology of Instituto Butantan that some *Bothrops moojeni* individuals injured by the same snake species showed mortalities caused by envenoming effects. This study analyzed the biochemical composition of 13 venom and plasma samples from *Bothrops moojeni* specimens to assess differences in their protein composition. Application of sodium dodecyl sulfate polyacrylamide gel electrophoresis (SDS-PAGE) showed distinct venom protein profiles, but very homogeneous plasma profiles. Western Blotting (WB) was performed with plasma samples, which were submitted to incubation with the respective venom. Some individuals showed an immunorecognized band zone around 25 kDa, indicating interaction between the same individual plasma and venom proteins. Crossed-WB assay using non-self-plasma and venom showed that this variability is due to venom protein composition instead of plasma composition. These venoms presented higher caseinolytic, collagenolytic and coagulant activities than the venoms without these regions recognized by WB. Mass spectrometry analyses performed on two individuals revealed that these individuals present, in addition to higher protein concentrations, other exclusive proteins in their composition. When these same two samples were tested *in vivo*, the results also showed higher lethality in these venoms, but lower hemorrhagic activity than in the venoms without these regions recognized by WB. In conclusion, some *Bothrops moojeni* specimens differ in venom composition, which may have implications in envenomation. Moreover, the high individual venom variability found in this species demonstrates the importance to work with individual analyses in studies involving intraspecific venom variability and venom evolution.

## Introduction

Snake venoms are probably one of the most complex of all known venoms, and are the most studied animal toxins since last century [1–4] to the present day [5–9]. Venoms are a highly effective adaptive trait underlying the evolution of advanced snakes, which have changed the way of prey capture from mechanical (constriction) to chemical (venom), and this innovation has played an important role in the diversification of these animals [10,11]. Snake venoms are subjected to Darwinian evolution [12,13] and the variability of these toxic secretions provides snakes with adaptability to different ecological niches [14]. The variability of venom composition and activities has been reported in numerous studies, and it can be observed at different levels, such as species, subspecies, populations, and individuals [15–20]. The composition of venoms results from multiple factors, and their inherent diversification is generally associated with environmental and ecological aspects [21].

Since the 18^th^ century, it is known that snakes are resistant to their own venoms. The first study addressing natural venom immunity was conducted by Fontana [22] in 1781, who reported that “the venom of the viper is not venomous to its species”. However, several concepts were further established in the 19^th^ century, including the one that the natural immunity is not species-specific [23] and can be found in other animals, such as several species of opossums, squirrels, mongooses, and hedgehogs [24–27]. This resistance is generally assigned to mutations in the gene encoding the target of the venom toxin, resulting in an insensitive target, and/or to the presence of neutralizing factors in the blood of resistant animals [28–30].

*Bothrops* species are responsible for over 90% of the notified snake accidents in Brazil [31], thus the epidemiology of this genus is of great medical and social importance [32]. Due to its clinical relevance, the most studied Brazilian snake species is *Bothrops jararaca*, and its venom and purified toxins have been comprehensively investigated. In addition, inhibitors from *B*. *jararaca* plasma and serum have been identified, isolated, and characterized, including phospholipase A_2_ inhibitors (PLIs) [33], a thrombin inhibitor that also binds to thrombin-like venom toxins [34,35], and a metalloproteinase inhibitor (the anti-hemorrhagic factor Bj46a) [30]. It is believed that these molecules play an important physiologic role in the protection mechanism against self-envenomation and envenomation by other snake species.

**Fig 1:**
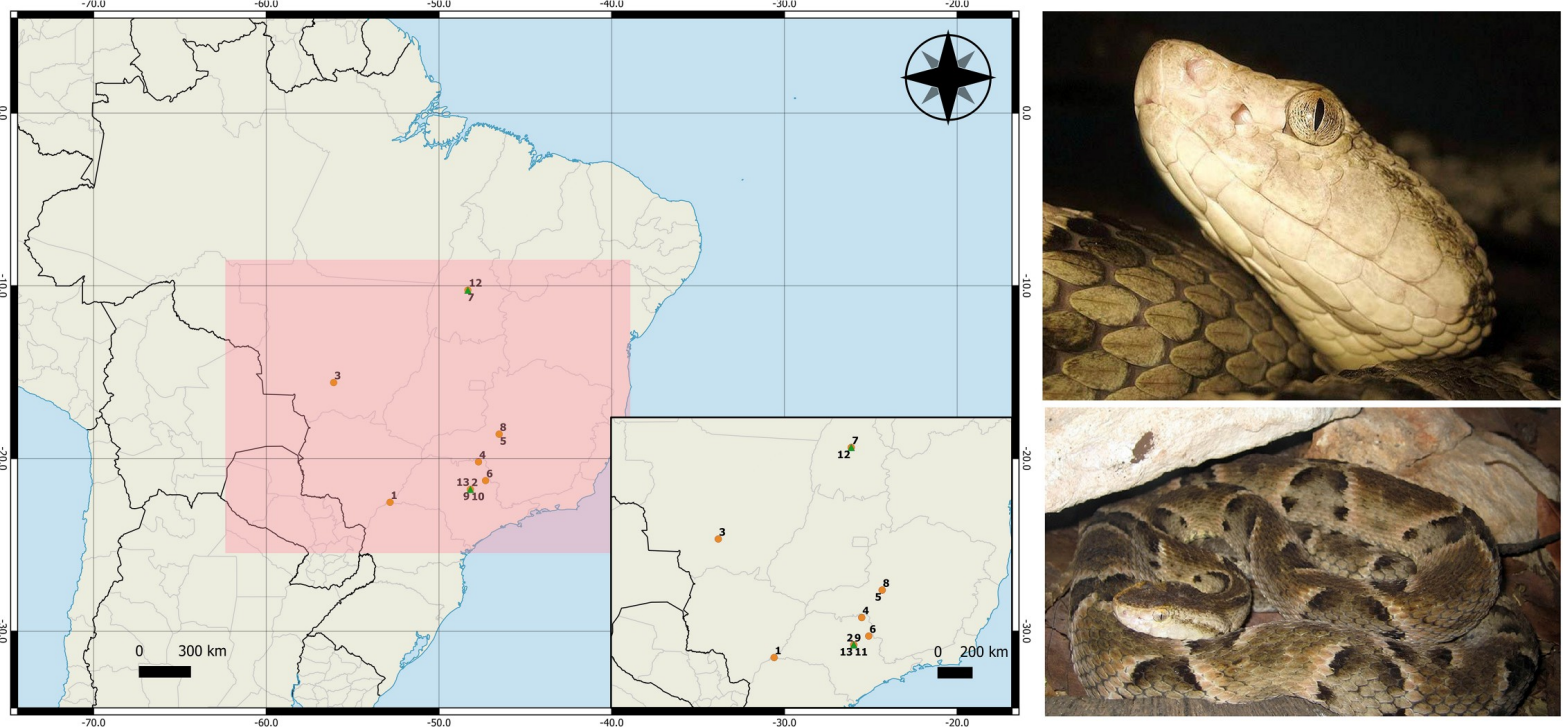
Geographic distribution of *B*. *jararaca* snakes used throughout this study. Orange dots represent female individuals and green triangles represent male ones.

Another species with high medical relevance in Brazil is *B*. *moojeni*, classified within Category 1 by the World Health Organization (WHO), corresponding to one of the species of highest medical importance, defined as “highly venomous snakes which are common or widespread and cause numerous snake-bites, resulting in high levels of morbidity, disability or mortality” [36]. This snake, popularly known as *caissaca*, is responsible for most of the snakebites in the Central region of Brazil (Fig 1) [37]. Its venom composes the pool of five *Bothrops* species, namely *B*. *jararaca* (50%), *B*. *alternatus* (12.5%), *B*. *jararacussu* (12.5%), *B*. *moojeni* (12.5%) and *B*. *neuwiedi* (12.5%), used to produce the bothropic polyvalent F(ab')2 antivenom (*soro antibotrópico*—SAB) by the Instituto Butantan (São Paulo, Brazil), using a method of hyperimmunization of horses. Due to the importance of the species from a medical standpoint, its venom proteome has been recently characterized. *B*. *moojeni* venom is composed mainly of snake venom metalloproteinases (SVMP) (36.5–39.8%), snake venom serine proteases (SVSP) (14.7-19-8%), phospholipases A_2_ (PLA_2_) (11.5–17.1%), L-amino acid oxidases (LAAO) (4.2–5.2%), and C-type lectins (CTL) (2.4–3.1%) Followed by less abundant toxins such as vascular endothelial growth factors (VEGF), bradykinin-potentiating peptides (BPP), cysteine-rich secretory protein (CRISP), and hyaluronidases, which together account for less than 15% of total venom proteins [38]. Reflecting its venom composition, the symptomatology caused by *B*. *moojeni* envenomation presents the classical local and systemic symptoms of bothropic accidents, such as edema, ecchymosis, necrosis, blisters, spontaneous bleeding, and blood incoagulability [39].

Concerning plasmatic venom inhibitors, only two molecules have been described in *B*. *moojeni* plasma to date, both of them are PLIs: a γ-PLI, identified by molecular biology [40], and an α-PLI (BmjMIP), isolated and characterized from its plasma [41]. Interestingly, BmjMIP presents the ability to neutralize enzymatic, toxic and pharmacological activities of basic and acidic PLA_2_ from *B*. *moojeni*, *B*. *pirajai* and *B*. *jararacussu* venom [41].

Given the background, the *B*. *moojeni* species represents an intriguing model, considering that a peculiarity observed in these animals is the high mortality caused by envenomation among individuals of this species, which is not observed in other snakes of this genus. In captivity, intraspecies envenoming accidents present visceral hemorrhage and necrosis in several spots, even in distal regions of the bite site (S1 Fig). This evidence indicates lack of a plasma defense mechanism against venom toxins and/or a particularity associated with individual venom composition. In summary, this comparative study describes the plasmatic venom inhibitors and the individual variability of venom composition and activities of 13 *B*. *moojeni* individuals.

## Material and methods

### Ethics statement

Swiss male mice weighing 18–22 g were obtained from the Center for Animal Breeding from Butantan Institute. Male and female individuals of *B*. *moojeni* snakes were obtained from the Laboratory of Herpetology of Instituto Butantan. All procedures involving animals were in accordance with the ethical principles for animal research adopted by the Brazilian Society of Animal Science and the National Brazilian Legislation n˚.11.794/08. After *in vivo* tests, mice were euthanized using a CO_2_ chamber, according to ethical parameters approved by the Brazilian College of Animal Experimentation (COBEA) and the Committee for the Ethical Use of Animals of Instituto Butantan (CEUAIB) (protocols n° 1304/14 and 1296/16).

### Snakes

Thirteen adult *B*. *moojeni* snakes, ten males and three females, were used in this present study. Information regarding the snakes selected for this study is available on Fig 1 and Table 1. The snakes are maintained under controlled temperature and light/dark cycles (12:12), in individual acrylic boxes, with free access to water and fed on rodents (*Mus musculus* and/or *Rattus novergicus*) once a month.

**Table 1:** Biometric data and geographical origin of the *Bothrops moojeni* specimens used throughout this study. Individuals were identified after venom and plasma extractions. Identification: code used in this study to identify the individual; In captivity since (year): year when the individual came from nature to captivity; Weight (g): weight of each individual when samples were collected; Length (SVL) (cm): length of the individuals when samples were collected. SVL means *snout-vent-length*; Geographical origin: the place where the individual came from; Sex: M: male; F: female.

Identification	In captivity since (year)	Weight (g)	Length (SVL) (cm)	Geographic origin	Sex
Bm1	2002	1,605	134	Porto Primavera—SP	F
Bm2	2006	1,78	146	Araraquara—SP	F
Bm3	2009	985	140	Cuiabá—MT	F
Bm4	2010	655	135	Buritizal—SP	F
Bm5	2010	1,175	138	Patos de Minas—MG	F
Bm6	2012	490	114	Cajuru—SP	F
Bm7	2008	1,865	168	Palmas—TO	F
Bm8	2013	990	132	Patos de Minas—MG	F
Bm9	2013	520	110	Araraquara—SP	F
Bm10	2012	345	109	Araraquara—SP	M
Bm11	2013	440	108	Araraquara—SP	M
Bm12	2005	1,08	138	Palmas—TO	F
Bm13	2013	300	111	Araraquara—SP	M

### Mice

Male Swiss mice were housed in a temperature-controlled room under automatic 12h light/dark cycle. Food and water were freely available during the study period. The experiments were performed using five animals per group, with a total of 110 mice for all experiments. After the tests, the animals were euthanized using a CO_2_ chamber, according to ethical parameters previously described.

### Plasmas and venoms

For venom milking of the 13 *B*. *moojeni* snakes, the specimens were individually anesthetized using a CO_2_ chamber. Venom samples were individually and manually collected, centrifuged at 1,700 x g for 15 min, lyophilized, and stored at -20° C until analyses. The *B*. *jararaca* venom pool, also obtained from the Laboratory of Herpetology of Instituto Butantan, which has been analyzed in a previous work [42], was used in some experiments for comparative purposes.

*B*. *moojeni* plasmas were obtained through blood collection from the same snakes in the presence of anticoagulant sodium citrate (3.8%) (9 volumes of plasma to 1 volume of anticoagulant) and centrifugation at 1,200 x g at room temperature for 15 min. Plasmas were stored at -20°C until use.

### Antivenom

Antibothropic antivenom produced by hyperimmunization of horses with a pool of five *Bothrops* species venoms, namely, *B*. *alternatus* (12.5%), *B*. *jararaca* (50%), *B*. *jararacussu* (12.5%), *B*. *moojeni* (12.5%), and *B*. *neuwiedi* (12.5%) was provided by the Plasma Processing Section of Butantan Institute, SP, Brazil (Batch 0712240/B).

### Compositional analyses of *B*. *moojeni* plasma and/or venom

#### Protein quantification

Protein concentration was assayed on individual plasmas and venoms according to the method described by Bradford [43] using the Bio-Rad Protein Assay reagent and bovine serum albumin (BSA) as standard. All samples were assayed in triplicate. Data were expressed as mean ±SDM.

#### Sodium dodecyl sulfate polyacrylamide gel electrophoresis *(*SDS-PAGE)

Venom and plasma samples were subjected to SDS-PAGE on 15 and 10% polyacrylamide gels, respectively, according to the method described by Laemmli [44]. Twenty micrograms of proteins were homogenized with sample buffer in the presence of 2-mercaptoethanol and applied to each lane. The polyacrylamide gels were stained using Coomassie Blue G250 according to the manufacturer's recommendations (GE Healthcare).

#### High performance liquid chromatography (HPLC)

Five hundred micrograms of lyophilized individual venoms were dissolved in 200 μL of 0.1% trifluoroacetic acid (TFA; solution A), centrifuged at 13,000 x g for 15 minutes, and separated by Reversed phase high performance liquid chromatography (RP-HPLC) using a Teknokroma Europa Protein 300 C18 column (0.46 cm x 25 cm, 5 mm particle size, 300 Å pore size). Elution was conducted at 1 mL/min by applying a gradient towards solution B (95% acetonitrile containing 0.1% TFA) according to Gay et al. [40] with some modifications: 5% B for 5 min, 5–25% B for 10 min, 25–45% B for 60 min, 45–70% B for 10 min, 70–100% B for 10 min, and 100% B for 10 min. Protein profile was detected at 215 nm.

#### Identification of plasma protein bands from SDS-PAGE

Protein bands from SDS-PAGE (S2 Fig) were excised and *in-gel* trypsin digestion (using 10 ng/μL sequencing-grade trypsin in 50 mM ammonium bicarbonate) (Sigma) was performed according to Hanna et al. [45]. Tryptic digests were dried in a SpeedVac (GeneVac) and stored at -20°C prior to LC-MS/MS analyses. Mass spectrometry experiments of venom digests were performed on a Synapt G2 HDMS (Waters) mass spectrometer coupled to a nanoAcquity UPLC system (Waters). Each peptide mixture was loaded online for 5 min at a flow rate of 8 μL/min of phase A (0.1% formic acid) using a Symmetry C18 trapping column (5 μm particles, 180 μm x 20 mm length; Waters). The mixture of trapped peptides was subsequently separated by elution with a gradient of 7–65% of phase B (0.1% formic acid in acetonitrile) through a BEH 130 C18 column (1.7 μm particles, 75 x 150 mm; Waters) in 20 min at 275 nL/min. Data were acquired in the data-independent mode MS^E^ in the m/z range of 50–2000 and resolution mode.

Collision energies were alternated between 4 eV (low energy) and a ramp of 19–45 eV (high energy) for precursor ions (MS) and fragment ions (MS/MS), respectively. Each MS and MS/MS step last 1 s, resulting in duty-cycles of 2 s. The electrospray ionization (ESI) source was operated in positive mode with a capillary voltage of 3.1 kV, block temperature of 100°C, and cone voltage of 35 V. For lock mass correction, a [Glu1]-Fibrinopeptide B solution (500 fmol/mL in 50% acetonitrile, 0.1% formic acid; Peptide 2.0) was infused through the reference sprayer at 500 nL/min and sampled every 60 s. Venom samples were analyzed in technical triplicates. Mass spectrometry raw data were processed in ProteinLynx Global Server 3.0.3 (Waters) platform using a low energy signal threshold of 750 counts and an elevated energy signal threshold of 50 counts. Database searches were performed against Serpentes (taxid: 8570) sequences from UniprotKB/Swissprot (www.uniprot.org; 2,608 reviewed sequences, downloaded on March 1^st^, 2019). The spectral deconvolution process for the MS^E^ experiments is described by Silva et al. [46]. Briefly, the algorithm cluster peptide components by accurate mass and retention time (AMRT) in the parallel low energy (MS1) and high-energy (MS2) acquisitions. The low-energy precursor ions are associated with their corresponding high-energy fragment ions by the obtained chromatographic attributes. The following search parameters were used: automatic tolerances for precursor and fragment ions, carbamidomethylation of cysteine residues as fixed modification, oxidation of methionine, N-terminal acetylation, glutamine and asparagine deamidation as variable modifications, and trypsin digestion with up to two missed cleavage sites allowed. Protein identifications were considered with a minimum of one fragment ion per peptide, five fragment ions per protein, two peptides per protein, and a false discovery identification rate set to 1%, estimated by simultaneous search against a reversed database [47,48].

#### Shotgun proteomics

Venom samples of 100 μg were dissolved in 50 μL of 50 mM ammonium bicarbonate, followed by addition of 25 μL of 0.2% RapiGest SF (Waters), and incubation at 80°C for 15 min. Samples were reduced with 5 mM dithiothreitol at 60°C for 30 min and then alkylated in the dark with 10 mM iodoacetamide at room temperature for 30 min. Proteins were digested using trypsin (Promega) at a 1:400 (wt:wt) enzyme:protein ratio at 37°C overnight. Digestion was stopped by addition of 10 μL of 5% trifluoroacetic acid and incubation at 37°C for 90 min. Samples were filtrated through 0.22 μm Millex-GV filters (EMD Millipore) into glass vials. The final protein concentration was approximately 2 μg/μL. Mass spectrometry experiments of venom digests were performed on a Synapt G2 HDMS (Waters) mass spectrometer coupled to a nanoAcquity UPLC system (Waters). Approximately 5 μg of each peptide mixture was loaded online for 5 min at a flow rate of 8 μL/min of phase A (0.1% formic acid) using a Symmetry C18 trapping column (5 μm particles, 180 μm x 20 mm length; Waters). The mixture of trapped peptides was subsequently separated by elution with a gradient of 7–35% of phase B (0.1% formic acid in acetonitrile) through a BEH 130 C18 column (1.7 μm particles, 75 x 150 mm; Waters) in 90 min at 325 nL/min. Data were acquired in the data-independent mode HDMS^E^ with ion mobility separation in the m/z range of 50–2000 and resolution mode. Collision energies were alternated between 4 eV and a ramp of 22–55 eV for precursor ion and fragment ions, respectively, using scan times of 1.0 s. The ESI source was operated in positive mode with a capillary voltage of 3.1 kV, block temperature of 100°C, and cone voltage of 35 V. For lock mass correction, a [Glu1]-Fibrinopeptide B solution (500 fmol/mL in 50% acetonitrile, 0.1 formic acid; Peptide 2.0) was infused through the reference sprayer at 500 nL/min and sampled every 60 s. Venom samples were analyzed in technical triplicates. Mass spectrometry raw data were processed in ProteinLynx Global Server 3.0.3 (Waters) platform using a low energy threshold of 750 counts and an elevated energy threshold of 50 counts. Database searches were performed against Serpentes sequences from UniprotKB/Swissprot (www.uniprot.org; 2,608 reviewed sequences, downloaded on March 1^st^, 2019). The following search parameters were used: automatic tolerances for precursor and fragment ions, carbamidomethylation of cysteine residues as fixed modification, oxidation of methionine, N-terminal acetylation, glutamine and asparagine deamidation as variable modifications, and trypsin digestion with up to two missed cleavage sites allowed. Protein identifications were considered only peptides with a minimum of 2 fragment ions per peptide, 5 fragment ions per protein and at least 2 peptides for protein, and a false discovery identification rate set to 1%, estimated by simultaneous search against a reversed database [47]. Label-free quantification was performed in ISOQuant [49] considering the averaged intensities of the three most intense peptides of each identified protein [50]. Significance of the differentially abundant proteins between the groups (with fold change ≥1.5 or ≤0.67) was determined using unpaired Student’s *t*-test considering *p*<0.05. The mass spectrometry proteomics data have been deposited to the ProteomeXchange Consortium via the PRIDE [51] partner repository with the dataset identifier PXD012585

### Functional analyses of *B*. *moojeni* plasma and/or venom

#### Western Blotting

The Western Blotting method [52] was used to verify the occurrence of protein binding between *B*. *moojeni* plasma and venom proteins. The proteins present in plasma, after SDS-PAGE separation, were transferred to a PVDF membrane (GE Healthcare) previously equilibrated in transfer buffer (25 mM Tris-base, 192 mM glycine, 20% ethanol) for 2 h, with a voltage of 20 V in a TE 77 PWR system (GE Healthcare). After transfer, the membrane was stained with Ponceau S to verify transfer efficiency (Ponceau 0.5% and acetic acid 1%) and destained with deionized water. After that, the membrane was cut to separate different specimen plasma lanes and then blocked with defatted milk (5%) containing 0.01% Tween 20 (incubation solution) and incubated overnight at 4°C. After three successive washes (5 min each) with washing solution (10 mM Tris, 150 mM NaCl, 0.01% Tween 20, pH 7.5), the membrane was incubated with the respective or non-self-venom (diluted 1:1,000) in incubation solution for 1 h at room temperature. The membrane was then incubated with antibothropic serum (Instituto Butantan) (diluted 1:1,000) for 2 h at room temperature. Immune-recognized proteins were detected using peroxidase-labeled for anti-horse IgG (diluted 1:10,000) and then revealed by adding the chromogenic substrate (5 mg of 3,3'diaminobenzidina tetrahydrochloride in 10 mL of imidazole buffer 0.1M, 125 μL 0.2 M CoCl_2_, and 3.4 μL 30% H_2_O_2_). Negative controls (data not show) were made with a membrane of only the plasma transferred from the SDS-PAGE and other with only individual venom samples.

#### Caseinolytic activity

Venom proteolytic activity on casein was determined as described by Menezes et al. [20]. Briefly, 10 μL of venom solution (1 mg/mL) and 500 μL of a solution of 2% N,N-dimethylated casein (Sigma), both solubilized in same buffer, were added to 490 μL of buffer (100 mM Tris-HCl, 10 mM CaCl_2_, pH 8.8). The mixture was incubated for 30 min at 37°C. The reaction was stopped by adding 1 mL of 5% trichloroacetic acid (TCA). The sample was then incubated for 10 min in ice bath, centrifuged at 14,000 x g at 4°C for 15 min, and absorbance of the supernatants was performed in a plate reader (Epoch–Biotek) using a wavelength of A_280_. Specific activity was expressed as units per minute per milligram of protein (U/min/mg).

#### Collagenolytic activity

Collagenolytic activity was determined as described by Vachova and Moravcova [53] and modified by Antunes et al. [54]. 6.25 μg of venom were incubated with 50 μL of 5 mg/mL azocoll (Sigma) solution, both resuspended in Tyrode buffer (137 mM NaCl, 2.7 mM KCl, 3.0 mM NaH_2_PO_4_, 10 mM HEPES, 5.6 mM dextrose, 1 mM MgCl_2_, 2 mM CaCl_2_, pH 7.4), in Thermo-shaker (Kasvi^®^) at 37°C and 1000 rpm for 1 h. The reaction was stopped by placing samples on ice. After centrifugation for 3 min at 5,000 x g, the absorbance of the supernatant (200 μL) was measured at 540 nm using a SpectraMax i3 microplate reader (Molecular Devices). One unit of activity was defined as the amount of venom that causes an increase of 0.003 units of absorbance, and specific activity was expressed as U/min/mg of venom. All samples were assayed in triplicate. Data were expressed as mean ±SDM.

#### Phospholipase A2 (PLA_2_) activity

The method based on the substrate 4-nitro-3-octanoloxy benzoic (NOBA) cleavage in the presence of phospholipase, releasing a chromogenic product that is measured at A_425_ [55], was employed to measure the PLA_2_ activity. Lyophilized venoms were diluted in 0.85% saline solution (1 mg/mL). In 96-well microplates (Sarstedt), 20 μL of venom samples were added to 20 μL of deionized water, 200 μL of 10 mM CaCl_2,_ 10 mM Tris buffer and 100 mM NaCl pH 8.0, and 20 μL of 3 mM NOBA (in acetonitrile). Next, the plate was incubated at 37°C for 20 min, followed by measuring of the A_425_. An increase of 0.1 A_425_ unit is equivalent to the release of 25.8 nmoles of chromophore. PLA_2_ activity was expressed as nmoles of chromophore released per minute of incubation per mg of protein (nmoles/min/mg).

#### L-Amino-acid oxidase (LAAO) activity

LAAO activity of venoms was determined using the method based on the amount of hydrogen peroxide released by the reaction between the LAAOs present in the venom and the reactive mixture [56]. Lyophilized venoms were diluted in 0.85% saline solution to a concentration of 0.5 mg/mL. Ten microliters of sample were incubated with 90 μL of reactive mixture (composed of 50 mM Tris-HCl, pH 8.0, 250 mM L-methionine, 2 mM o-phenylendiamine (OPD), and horseradish peroxidase 0.8 U/mg) in microplates (Sarsted) at 37°C for 1 h. The reaction was then stopped with 50 μL of 2 M H_2_SO_4_ and read at A_492_ in a plate reader (Epoch–Biotek). Determination of the LAAO activity was performed using a standard curve of hydrogen peroxide. Results were expressed as μM of H_2_O_2_ produced/min/mg of venom (nM/mg/min).

#### Coagulant activity

Minimum Coagulant Dose (MDC) was defined as the smallest amount of venom that clotted a standardized citrated plasma or fibrinogen solution in 60 seconds at 37°C [57].

MCD was determined in samples of citrated bovine plasma (MCD-P) and bovine fibrinogen solution (2 mg/mL) (MCD-F), as described by Theakston and Reid [57]. Venoms were diluted in 0.85% saline solution in concentrations ranging from 1.95 to 1,000 μg/mL. Clotting time was obtained by adding 100 μL of each venom dilution to 200 μL of plasma or fibrinogen, maintained at 37°C. Coagulation time was recorded immediately after addition of the venom dilution to the coagulometer (Drake).

#### Hemorrhagic activity

Hemorrhagic activity was measured as previously described [58] and modified by Gutiérrez et. al. [59]. Five groups of five male Swiss mice weighing 18–22 g were injected intradermally in the abdominal region with different doses of venom (2.5, 5, 10, 20 and 30 μg/mL) in volumes of 100 μL. After 2 hours of inoculation, mice were euthanized using a CO_2_ chamber. The skin of the ventral region was removed and fixed on a glass plate. The hemorrhagic area was transferred to a white paper sheet, subsequently digitized, and measured using the ImageJ software. The minimum hemorrhagic dose (MHD) was defined as the amount of venom that produces a 10 mm diameter hemorrhagic halo.

#### Lethal dose

Median lethal dose (LD_50_) was defined as the dose responsible for the death of 50% of the population analyzed [60]. Different doses of venom (44.4, 66.6, 100, 150 and 225 μg/mL) were resuspended in 500 μL of sterile 0.9% saline solution and injected intraperitoneally in the male Swiss mice (18–22 g). Five animals were used for each venom dose (55 animals– 25 per group, including one control group). Deaths were registered during a 48 h period (4h interval, approximately), and the LD_50_ was estimated by Probit analyses [61]. Remaining mice were euthanized utilizing previously described protocol.

## Statistical analysis

Data were expressed with median and ± standard deviation (SD). Results were analyzed with one-way ANOVA, with Tukey as a posteriori test and values of p<0.05 was considered significant.

## Results and discussion

### Compositional and functional analyses of *B*. *moojeni* plasma and venom

In this work, we used an unbalanced sex distribution of individuals (only three males to ten females) since we were bounded to the individuals available for this study in our laboratory. At that time, we have other male individuals from the same region (Araraquara - SP) and that would not increase our range of individuals from different regions, as we had with the female distribution. We also did not used any juvenile individuals in our analysis, since all the cases of cross envenomation observed by our group was among adult individuals only, during mate season. From this statement, our results will now be discussed below.

**Fig 2:**
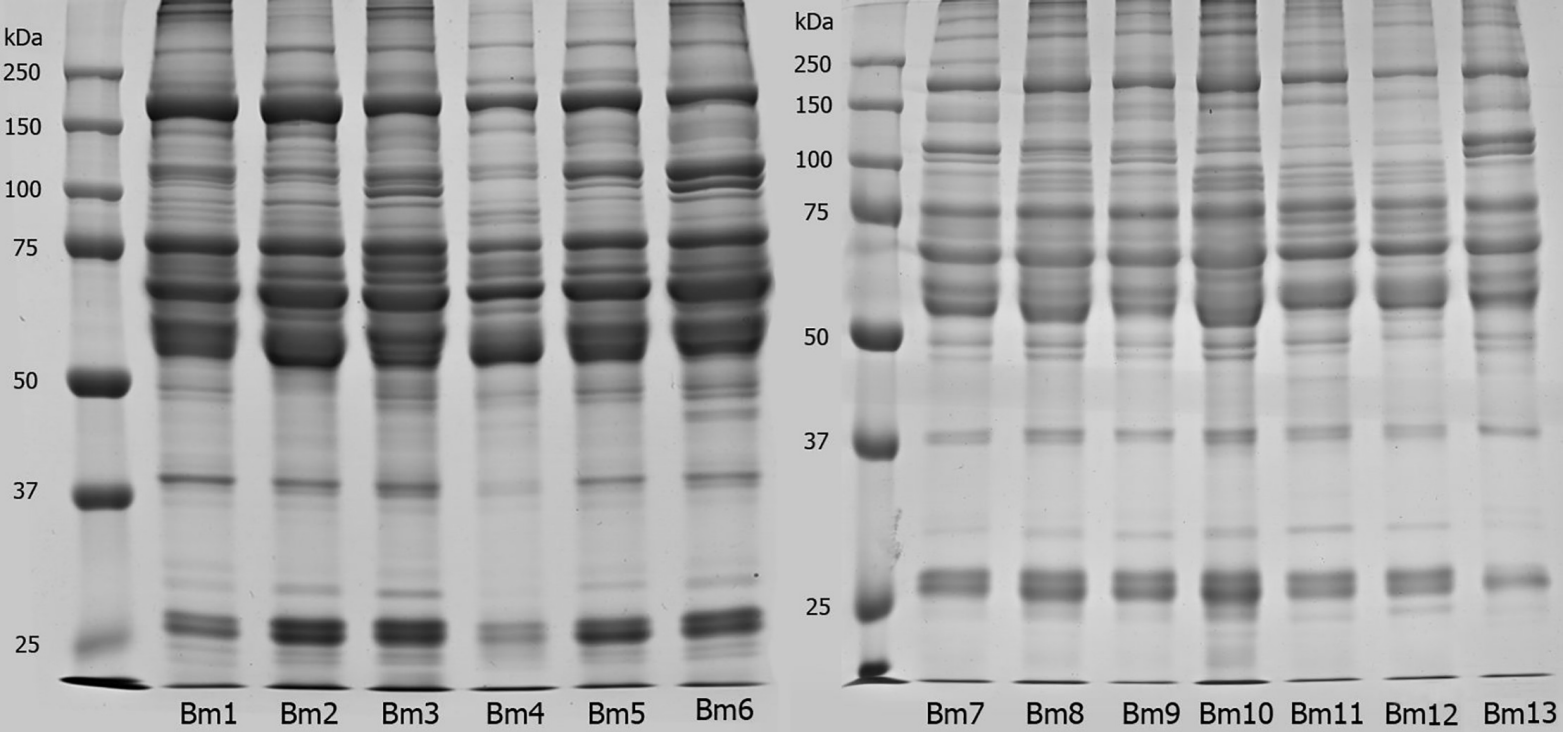
Protein profile of *B*. *moojeni* plasma samples. *B*. *moojeni* plasma samples (20 μg) were individually analyzed by SDS-PAGE (10%) under reducing conditions. Gels were stained with Coomassie Blue G250. Lanes correspond to individual plasmas from specimens Bm1 to Bm13.

The protein profile of individual plasmas was analyzed by 10% SDS-PAGE under reducing condition. Fig 2 shows the electrophoretic profile of *B*. *moojeni* snake plasmas, numbered from 1 to 13.

Patterns of plasma proteins showed similarity between all samples, with slight differences in the intensity of few bands, especially in bands with ~30 kDa and ~37 kDa. Although no exclusive bands were visually identified, it was possible to notice that some points, such as the region immediately below the 50 kDa in individuals 6 and 11, displayed a well-defined band that barely appeared in others. Between 50–75 kDa, three intense bands and bands with different concentration were observed in all individuals, showing some degree of variability. Snake plasma composition of the *Bothrops* genus includes, but it is not limited to, gamma-type PLA_2_ inhibitors (at the 20~30 kDa range), transferrins, antihemorragic factor, and albumin (50~150 kDa) [18], a myotoxin inhibitor that has already been described with 25 kDa [41] and Bj46a, which is a glycoprotein that inhibits metalloproteinases and has been previously found in *B*. *jararaca* [62].

**Fig 3:**
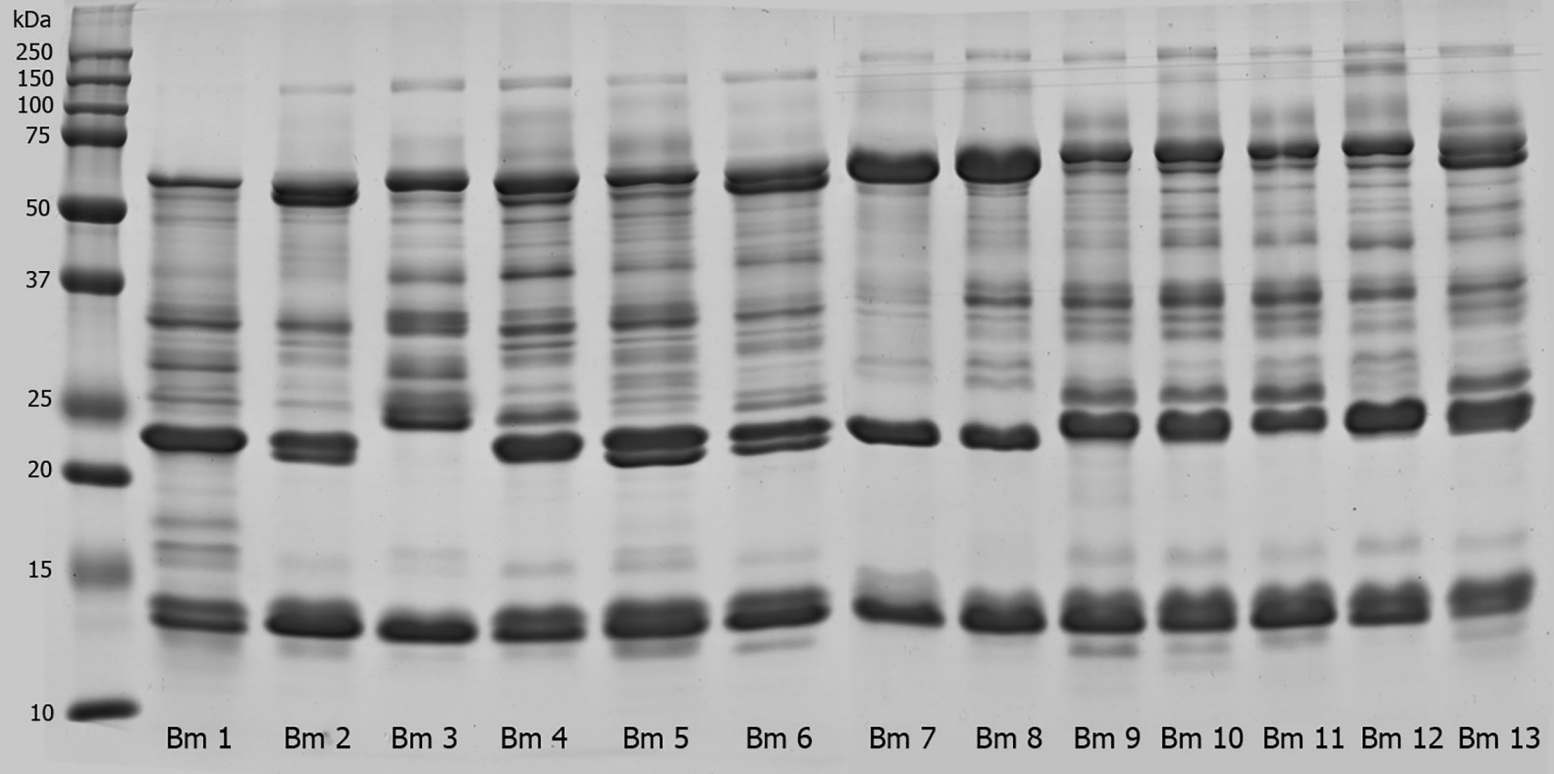
Protein profile of *B*. *moojeni* venom samples. *B*. *moojeni* venom samples (20 μg) were individually analyzed by SDS-PAGE (15%) under reducing conditions. Gels were stained with Coomassie Blue G250. Lanes correspond to individual plasmas from specimens Bm1 to Bm13.

Protein bands were assigned to the major protein families according to previously studies. The profile of individual venom samples was analyzed by 15% SDS-PAGE under reducing condition. As shown in Fig 3, the electrophoretic profile of *B*. *moojeni* snake venoms showed a remarkable intraspecific variation between specimens. First, individual 3 showed a profile distinct from the others, with lack of a band in the 20–25 kDa region and higher variability in the 25–37 kDa range of the gel. Some individuals showed similar SDS-PAGE profile, as in the case of individuals 9, 10, 11, and 13, all originally from the same region (Table 1). These four specimens were brought to Instituto Butantan in 2013, except the individual 10, which arrived a year earlier. Therefore, it is interesting to point out that, even with different captivity periods, these four individuals from the same geographic region still presented similar protein profiles. All individuals showed a band with ~100–150 kDa, except individual 1. This region corresponds to phosphodiesterases (PDE) and/or prothrombin activator subunits [63,64]. The region between ~50–75 kDa presented a major protein band in all individuals. This band corresponds to snake venom metalloproteinases class P-III (SVMP-III), one of the main groups of toxins of genus *Bothrops* venoms [63,65,66]. It was also possible to note that this area presents two bands instead of only one in individuals 4, 6, and 13. A band with 37 kDa was observed in all venoms, except in those of individuals 2 and 7. The venoms showed a variable band composition in the range of 25–37 kDa, displaying at least five protein bands. In contrast, individual 7 presented less intense bands in this region. Specifically, individuals 2, 7, 8, and 12 lack a band in the 25 kDa region, whereas individual 13 presented the most intense 25 kDa band. The main components assigned to molecular range between 25–37 kDa are snake venom serine proteases (SVSP) and snake venom metalloproteinases class P-I and P-II (SVMP-I and SVMP-II) [65]. The most intense band in all samples was located between 20 and 25 kDa. Surprisingly, individual 3 did not present this band, whereas individuals 2, 5, and 6 markedly showed two bands. Finally, the region between 10 and 15 kDa, associated with PLAs [67], showed a slight variation between the samples. All individuals exhibited two intense bands in this region, except individuals 3 and 7, which showed only one intense band. The profile of individual 7 is different from that of individual 12, although both came from the same region, but in different years (seven in 2008 and 12 in 2005). These results show the high variability in venom composition between individuals of this species, as well as compared with other species previously mentioned by other authors [16,37,68]. In fact, snake venoms are the result of a series of adaptations in their composition in order to make the best protein mixture to both type of prey and the environment in which snakes are inserted [69]. In addition, the quantitative and qualitative composition of the venoms are associated with several factors such as habitats, gender, age, and nutrition [37,70–72], and cannot be directly related to one single factor.

**Fig 4:**
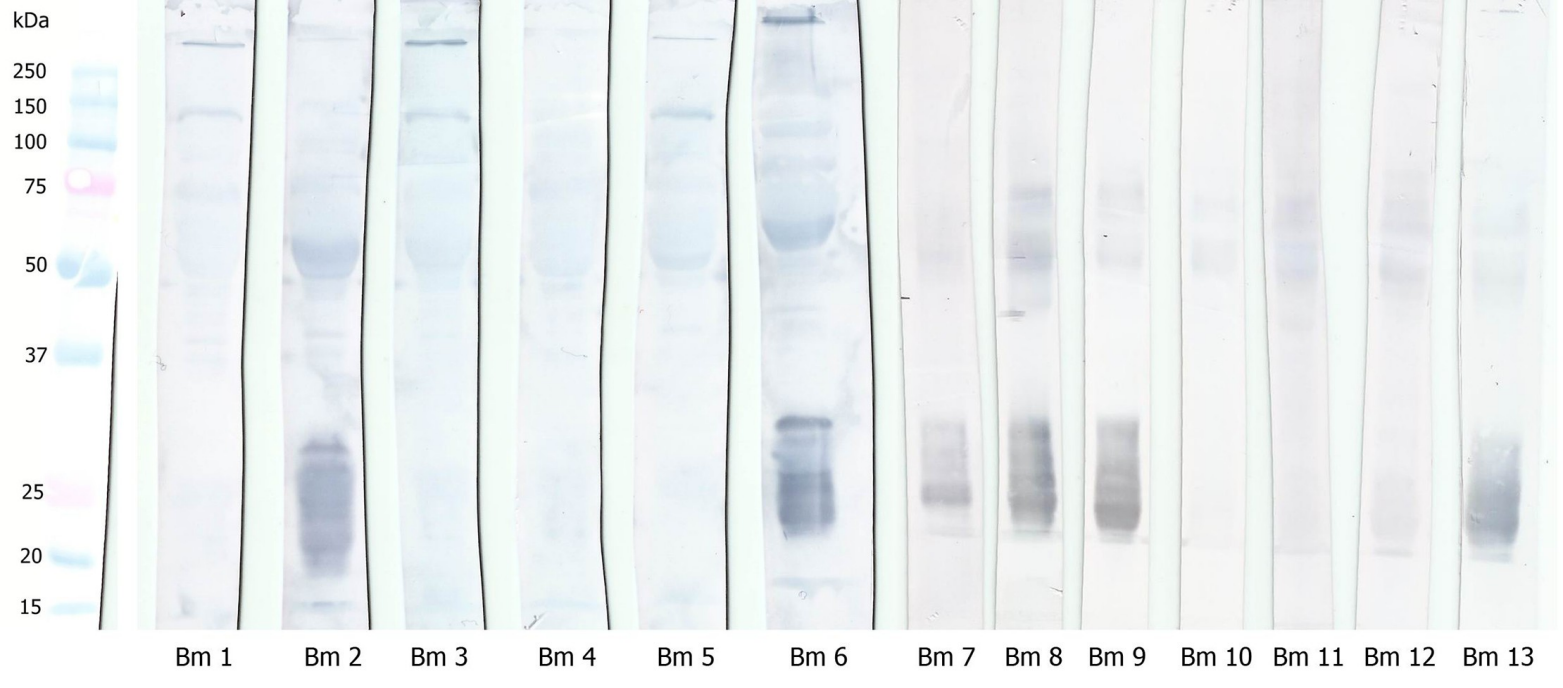
Interaction between *B*. *moojeni* plasma and venom proteins evaluated by Western blotting. *B*. *moojeni* plasma proteins (20 μg of protein) were fractionated by SDS-PAGE (10%, under reducing conditions), electrotransferred onto PVDF membranes, and incubated with the respective venom, prior to the incubation step with antibothropic serum.

As many different profiles were found, we decided to subject all individual venom samples to the WB technique so that the interaction between plasmatic proteins and their respective venom could be analyzed. As shown in Fig 4, there are peculiar binding regions in the range of 20–30 kDa of plasma proteins, indicating interaction between plasma and venom proteins in individuals 2, 6, 7, 8, 9, and 13.

**Fig 5:**
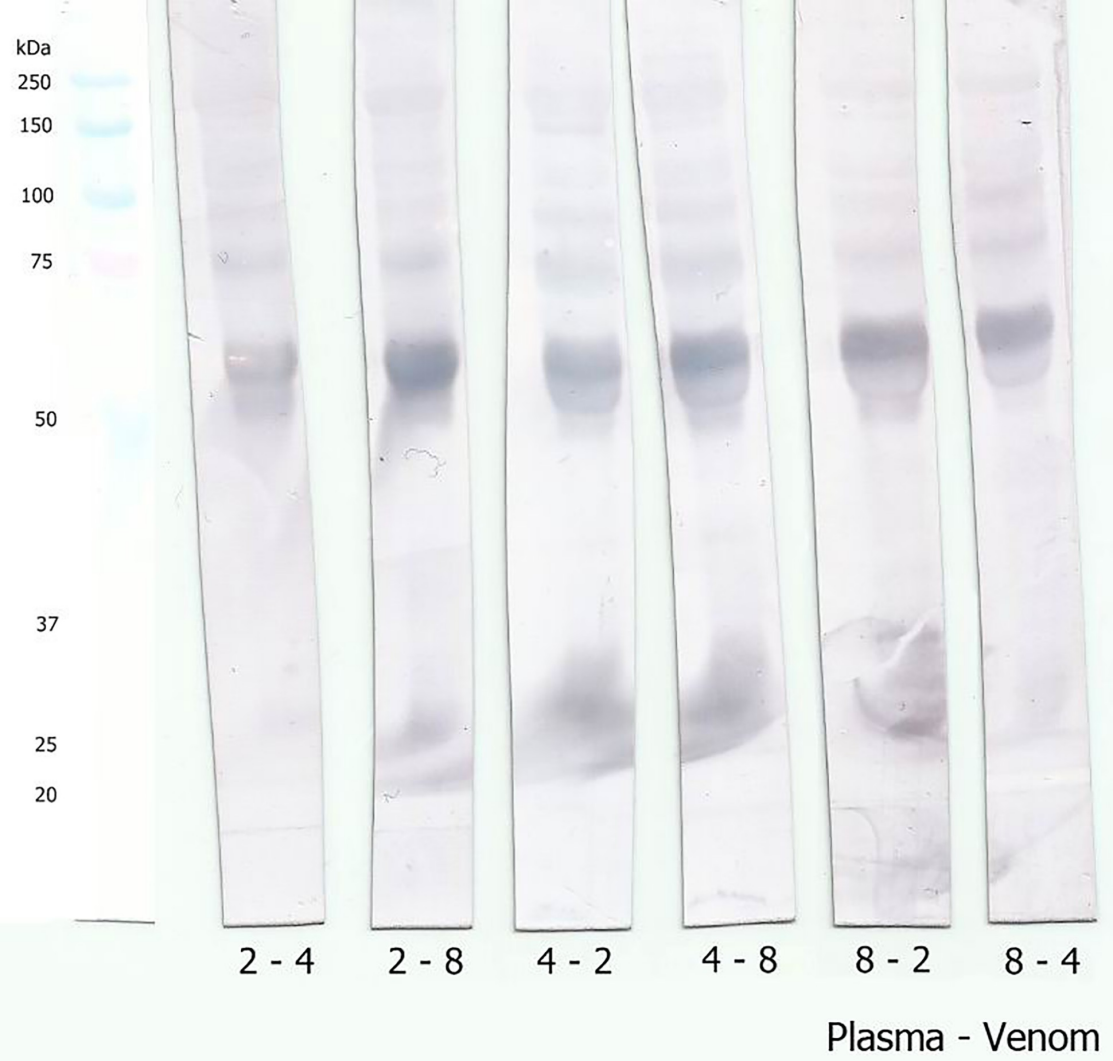
Cross-reactivity between plasmas and venoms from different specimens of *B*. *moojeni*. *B*. *moojeni* plasma proteins from specimens Bm2, Bm4, and Bm8 (20 μg of protein) were fractionated by SDS-PAGE (10%, under reducing conditions), electrotransferred onto PVDF membranes, and incubated with the non-self venom prior to the incubation step with antibothropic serum. The first number below the membrane strips corresponds to the plasma specimen number and the second number corresponds to the venom specimen number.

In order to analyze whether there was cross-reactivity between plasma and venom proteins from different specimens of *B*. *moojeni*, another set of WB was performed. Three individuals were selected for these experiments: two venom samples from individuals that presented that interaction (specimens Bm2 and Bm8) and one sample from an individual that did not show the interaction (specimen Bm4). Results revealed that the plasma sample from the specimen that previously did not present a recognition area in the 20–30 kDa region, when incubated with venom from a specimen (Bm2 or Bm8) that showed the previously immune interaction, also exhibited the recognition reaction in this region (Fig 5). This finding suggests that the differences on the recognition pattern observed by WB are associated with venom composition, considering that the venom of an individual that interacts with its own plasma is also able to interact with plasma from other individuals.

The plasma proteins with ~25 kDa involved in the recognition of venom proteins were identified by mass spectrometry after in gel trypsin digestion. Results (S2 Fig and S1 Table) showed that these protein bands correspond mainly to PLA_2_ inhibitors, as previously described in *B*. *moojeni* plasma [41]. In addition, the anti-hemorrhagic factor BJ46a (FTE46_BOTJA), a molecule originally described in *B*. *jararaca* plasma [62], was also identified.

### RP-HPLC

**Fig 6:**
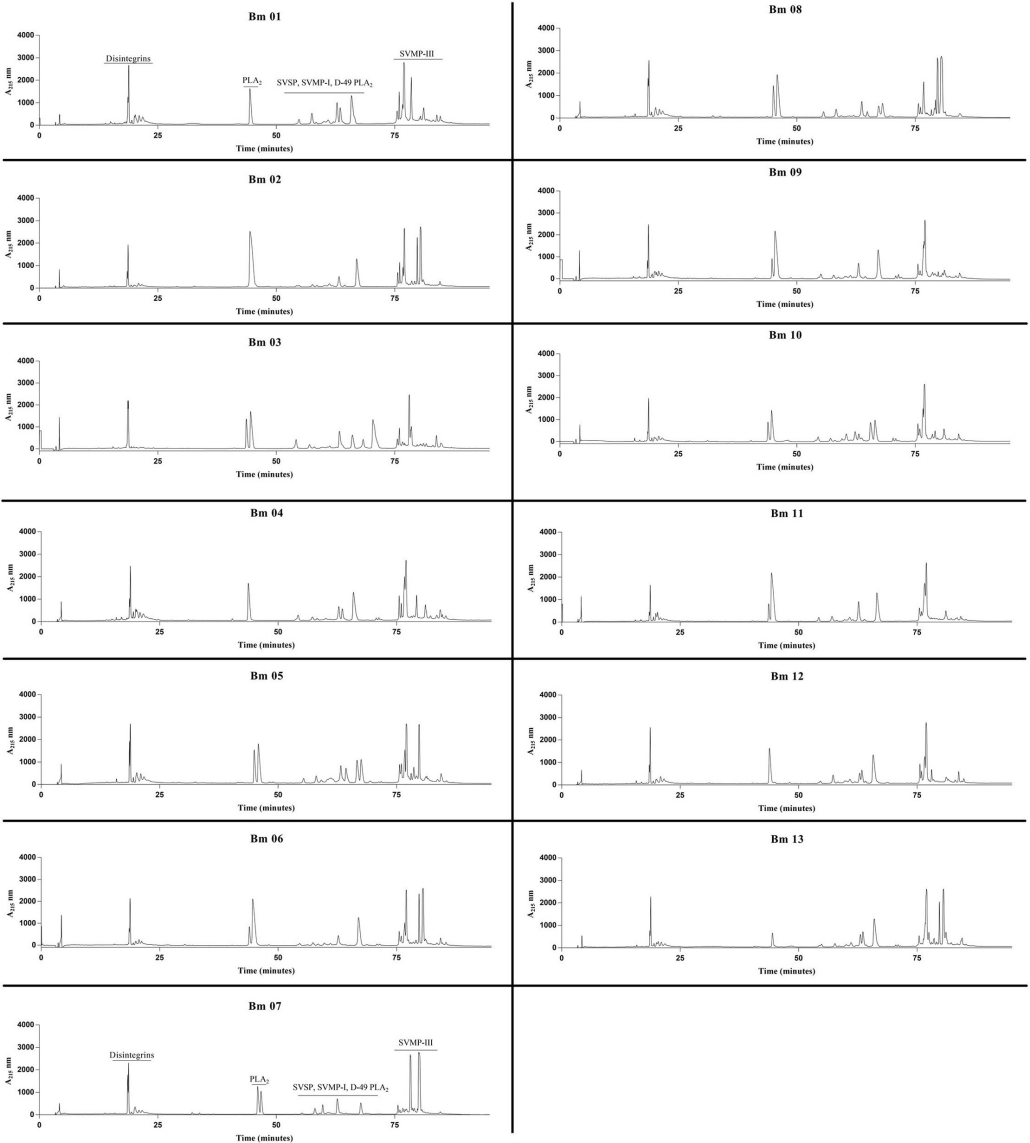
Elution profiles of *B*. *moojeni* venom proteins by RP-HPLC. Samples of 500 μg of lyophilized venoms from specimens Bm1 to Bm13 were dissolved in 0.1% trifluoroacetic acid (TFA; solution A) and subjected to RP-HPLC in a C18 column. Elution was conducted at 1 mL/min by applying a gradient towards solution B (95% acetonitrile containing 0.1% TFA) as follows: 5% B for 5 min, 5–25% B for 10 min, 25–45% B for 60 min, 45–70% B for 10 min, 70–100% B for 10 min, and 100% B for 10 min. Absorbance was measured at 215 nm.

The protein profiles of all thirteen individuals were evaluated by RP-HPLC in a C18 column. In general, results showed a typical bothropic chromatographic venom profile (Fig 6), but also evidenced differences regarding peak intensity in some samples. Using a previous report as a proteomic reference [73], the peaks eluted before 25 min are mainly composed of disintegrins, K-49 PLA_2_ are eluted after ~45 min mark, SVSP, SVMP-I, D-49 PLA_2_, and LAAO are grouped in different peaks between 50–75 min, and SVMP-III predominating after the 75 min mark. A difference in the K-49 PLA_2_ region was observed when the individual HPLC profiles were compared. This species seemed to present two profiles in this area: one showed a single peak (individuals 1, 2, 4, 12, and 13) and the other presented two distinct peaks (individuals 3, 5, 6, 7, 8, 9, 10, and 11). Individual 13 presented a small peak in this area. A variable region was also observed around 60 min, which comprises several major protein families. The SVMP region showed variable profiles between individuals as well. The individuals presented between one and four peaks in this area. Results also showed that even the individuals of the same sex or from the same region exhibit variable chromatographic profiles, with quantitative and qualitative differences. However, this higher individual variability was expected because the first analyses and results of this study corroborated those of other studies [74–76]. Since it was in our best interest to identify the differences found previously, we decided for two individuals that represented, so to speak, the two different ‘groups’ that we identify with the WB test. With that in mind, the individuals Bm2 and Bm4 were selected to be used on a more thorough analysis. For the *in vivo* assays and to be analyzed by Quadrupole time-of-flight—Nanoflow electrospray-ionization (QTOF-nanoESI). With that in mind, we will discuss all the following results focusing specifically on these two individuals.

### Shotgun proteomics

**Fig 7:**
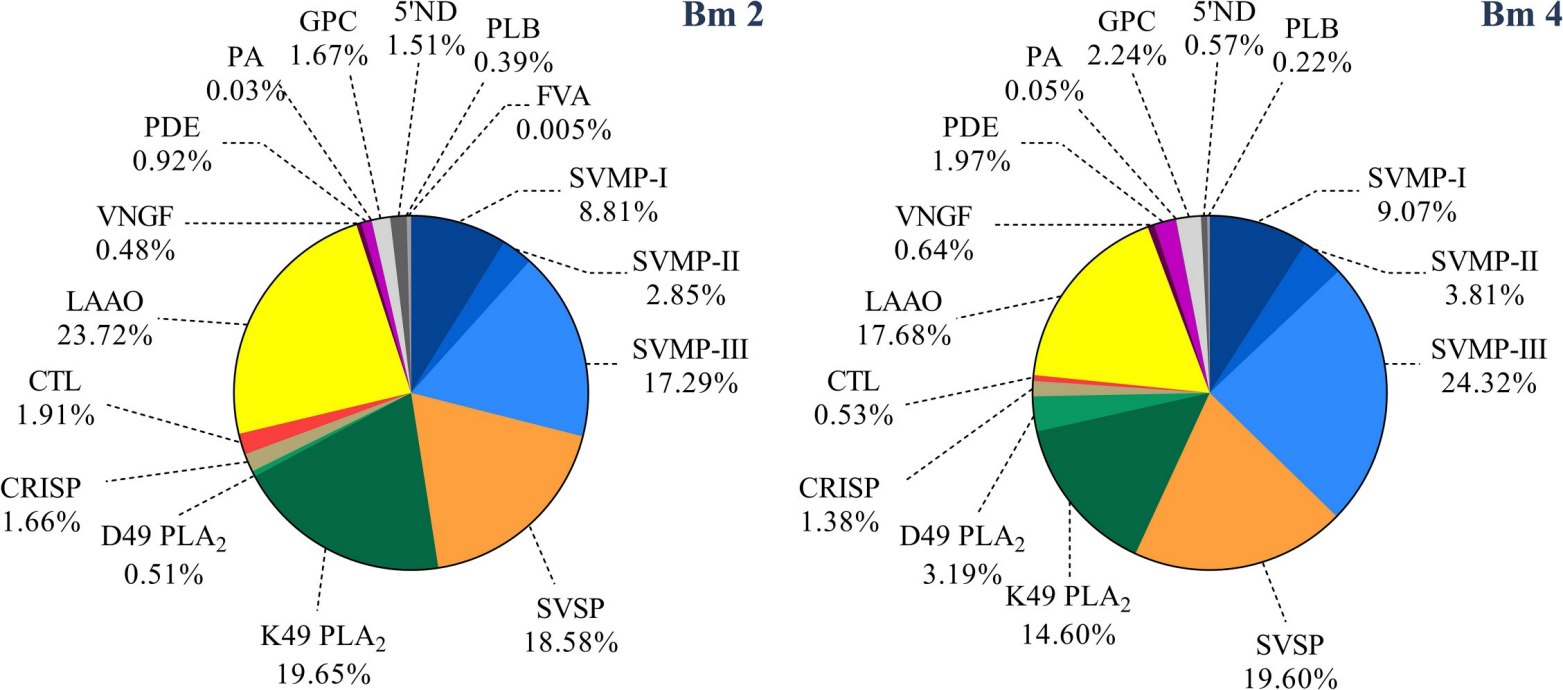
Overall composition of *B*. *moojeni* snake venom. Individuals Bm2 and Bm4, according to protein families, expressed as percentages. (PI-, PII- and PIII-SVMP) snake venom metalloproteinase of PI, PII and PIII class, respectively; (SVSP) snake venom serine protease; (D49/K49 PLA_2_) phospholipase A_2_ D49 and 49, respectively; (CRISP) Cysteine-rich secretory protein; (5’ND) 5’Nucleotidase; (PLB) phospholipase B; (GPC) Glutaminyl-peptide cyclotransferase; (CTL) C-type lectin; (PDE) phosphodiesterase; (LAAO) L-amino acid oxidase; (VNGF) venom nerve growth factor; (PrAct) prothrombin activator; (PlasmAct) plasminogen activator.

Protein composition of Bm2 and Bm4 was determined by quantitative shotgun proteomics. In the search engine ProteinLynx Global Server (PLGS), we have the option to set mass error tolerances in automatic mode or to define a fixed mass error. In the automatic mode, PLGS calculates the appropriate tolerances based on the error distribution of the PSM identifications. We see from our data that most PSM are within the 10 ppm mass window and none outside the 40 ppm window. The results (S1 Table) showed identification of one exclusive protein in the venom of Bm2 and nine in Bm4. A basic PLA_2_ Lys-49 (Accession code: Q9I834) was the only exclusive protein found in the Bm2 venom. This protein lacks enzymatic activity (due to the presence of a lysine at position 49) but display myotoxicity and edema-inducing activity [77]. Bm4 also have an exclusive PLA_2_ with the same characteristics (Q9PVE3), and other one that shows enzymatic activity (P0C8M1). Besides that, this individual also presented three metalloproteases; one with weak hemorrhagic activity (P83512), one PII type (O57413) and one PIII type (Q9DGB9). Other three serineproteases were also find in its composition: one thrombin-like that does not coagulate human plasma (P81176), other thrombin-like with fibrinogenolytic activity *in vitro* and defibrinogenating *in vivo* (K4LLQ2) and the last one that plays a role in the hemostasis system on the prey (Q5W960). Together, these two individuals share a total of 60 common proteins. Regarding the families of those common proteins, these compounds belong to PLA_2_, C-type lectin, LAAOs, metalloproteinases, serine proteases, CRISPS, Glutaminyl-peptide cyclotransferases (GPC), and neural growth factors. Fig 7 shows a quantitative distribution of the compounds present in both individuals. It could be possible that the exclusive PLA_2_ found in Bm2 is the one that reacts with the plasma samples during the Western blotting assay, since that plasma region is composed mostly by gamma-PLI inhibitor (S2 Table).

The abundant amount of serine proteases (14.41% in Bm2 and 17.94% in Bm4) and type III metalloproteinases (20.24% in Bm2 and 25.53% in Bm4) was expected considering that *B*. *moojeni* is well known for its higher proteolytic and hemorrhagic activities [78]. That may be the cause of edema and generalized hemorrhage previously reported in the *B*. *moojeni* autopsy records of individuals in our laboratory. There have been reported some cases of death suggestive of envenomation by bites by other individuals during the mating season, when these animals were grouped to copulate (in captivity). The autopsy records described anomalies registered as “hemorrhage regions in the ventral and distal areas”, even when the bite had occurred in a distant region, such as the tail or head of the animal (S1 Fig). The bothropic venom is well known for its ability to cause local tissue damage and other proteolytic disturbances such as hemorrhage and/or coagulation. It is worth noting that snakes, along with some of their prey and predators, have “protecting factors”, known as inhibitors, which protect them from the effects of the many different toxic proteins present in their composition. These intriguing cases of death by envenomation can be associated with the high variability that these individuals present in their venom composition, as well as with the analyses of the specific activities that will be described ahead.

### Functional analyses of *B*. *moojeni* venom

#### Caseinolytic activity

**Fig 8:**
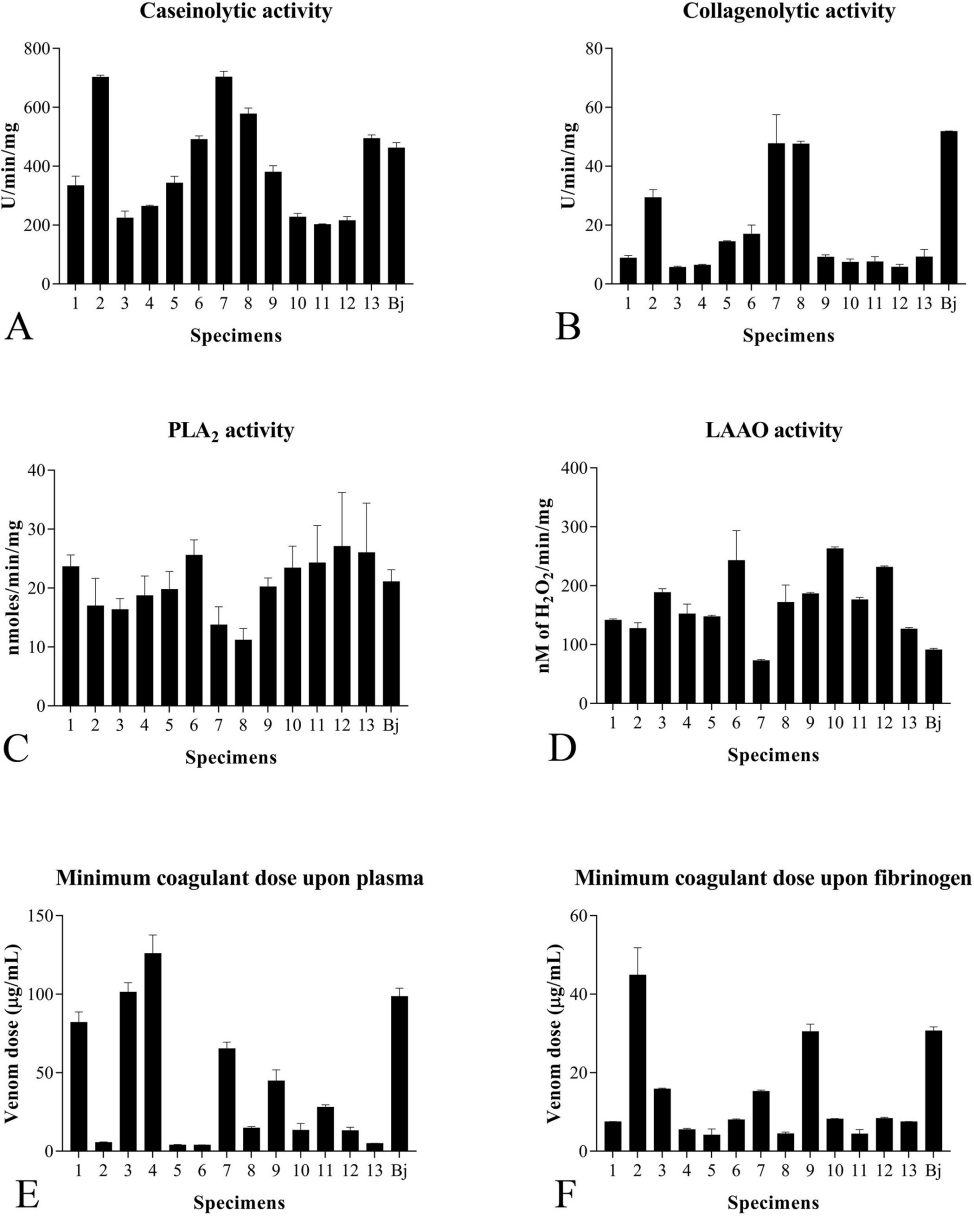
Enzymatic activities of *B*. *moojeni* individual venoms. Results of biological characterization assays of all 13 *B*. *moojeni* and one *B*. *jararaca* individuals. Collagenolytic activity (A); caseinolytic activity (B); PLA_2_ activity (C); LAAO activity (D); MCD-Plasma activity (E); MCD-Fibrinogen activity (F).

Venom proteolytic activity using casein as substrate showed a diverse profile between all 13 individuals (Fig 8A). The highest activity was from individual Bm2, with 702.22 ±6.94 U/min/mg, whereas the lowest one was from individual Bm11, with 202.22 ±1.92 U/min/mg. In addition, individual Bm2 is statistically different from all other ones, while Bm4 did not showed statistical difference with individuals Bm3, 10 and 12.

#### Collagenolytic activity

Using collagen to evaluate the proteolytic activity, a pattern different from that described using casein as substrate was observed. Interestingly, the highest caseinolytic activities were found in individuals whose venom showed plasma interaction (Fig 8B). Statistically, individual Bm2 showed the same results from caseinolytic activity, while Bm4 differed from the samples Bm6, 7, 8 and Bj. In addition, these results are in agreement with those obtained in the chromatography profiles: when an individual showed a distinct second and/or third peak after the 75 min mark, its proteolytic activity presented a result higher than those of other individuals, suggesting the presence of a collagen-specific protease in this fraction. Curiously, our MS results showed a different pattern between individuals Bm2 and Bm4. While the second one presented a higher total concentration of proteases, its proteolytic results were lower than the Bm2. Roodt et al. previously reported that *B*. *moojeni* presents higher proteolytic activity compared with that of other snakes from the *Bothrops* genus [79]. In this study, *B*. *jararaca* venom, used as control, showed a caseinolytic activity of 462.22 U/min/mg, approximately 35% lower than the value of 703.99 U/min/mg obtained from the individual with the highest caseinolytic activity that positively reacted with plasma, as demonstrated by the WB test. Curiously, in the collagenolytic assay, different pattern was observed, in which the *B*. *jararaca* sample showed the highest collagenolytic activity among all samples tested. These results demonstrate that, differently from *B*. *moojeni* proteases, *B*. *jararaca* proteases present higher specificity to collagen than to casein.

#### PLA_2_ activity

Concerning the PLA_2_ activity of individual venoms, different results were obtained between the samples, ranging from 11.18 ±1.97 to 27.09 ±9.14 nmols/min/mg (Fig 8C). Statistics showed no significant differences amongst all samples, with the exception of Bm6 (different from Bm8) and Bm8 (different from Bm6, 12 and 13).

#### LAAO activity

LAAO activity also showed high individual variations. Statistics confirm this affirmation, with all individuals showing differences between them all (p<0.05). The values obtained for this enzymatic activity ranged from 72.73 ±2.02 to 262.98 ±2.86 nM/μg/min (Fig 8D). Mass spectrometry showed a different result, with individual Bm4 having a higher activity (against Bm2), despite its lower LAAO abundance (17.68%) in comparison with Bm2 (23.72%).

Campos and colleagues [80] also compared the PLA_2_ and LAAO activities between the venoms of *B*. *moojeni* and those of other snakes of the *Bothrops* genus and concluded that the venom of the first presents higher activities than those of the latter. These data support the present study, in which more than 50% of the *B*. *moojeni* individuals showed increased PLA_2_ activity compared with that of *B*. *jararaca*. Regarding the LAAO activity, all *B*. *moojeni* individuals presented higher activity than *B*. *jararaca* individuals, as depicted in Fig 8. While mass spectrometry results confirm the PLA_2_ results, in which the individual Bm4 showed a higher specific activity when compared to the Bm2 individual, the LAAO results showed an inverse result when comparing the same two individuals (Fig 7). It is known that the LAAOs act as adjuvants in envenoming, in which the H_2_O_2_ released causes a cytotoxic effect by increasing the action of other proteins present in the venom. They are responsible for some different biological activities, including cytotoxicity, tissue necrosis, apoptosis induction, and platelet aggregation inhibition, causing innumerous hemostatic disorders. All these activities are closely related to the cases of bleeding, important in the envenoming process [81–84]. PLA_2_ activities, which act in the lipid bilayer deconstructing the cell membrane, also have activities such as neurotoxic effects, myotoxicity, inhibition of platelet aggregation, edema, hemolysis, anticoagulation, convulsion, and hypotension [85–87].

#### Minimum coagulant dose

Both substrates used in this experiment (plasma and fibrinogen) showed high individual variability between all samples (Fig 8E and 8F). Regarding coagulation on plasma, individual Bm6 presented the lowest MCD (3.9 ±0.10 μg/mL) and individual Bm4 showed the highest MCD (125.99 ±11.72 μg/mL) on the same substrate. With respect to fibrinogen, individuals Bm2 and Bm5 presented the highest and lowest MCD, 44.85 ±6.97 and 4.14 ±1.55 μg/mL, respectively. In addition, individual Bm2, which was used alongside with individual Bm4 in the *in vivo* assays, showed a low MCD-P (5.57 ±0.32) and a high MCD-F (44.85 ±6.97). Statistically, individuals Bm10, 12 and 13 showed differences only with *B*. *jararaca*. Both individuals analyzed by mass spectrometry have a similar abundance of SVSP (Bm2 18.25%; Bm4 19.60%) in its composition. It is important to point out that the plasma used in this assay was not recalcified to match its original physiological state. This lack of recalcification may have influenced the relative potency of those venoms, altering its original coagulation times, since it is known by recent studies that snakes from the *Bothrops* genus are highly calcium dependent when it comes to coagulation properties [88]. There might be some variation in calcium dependence state among different individuals, but we could not observe that in this study. So, for future references, our group will perform coagulations assays with recalcified plasma only. Therefore, this coagulation data shall be compared only to results from other works that also did not recalcified their substrates as well.

The two individuals analyzed showed an inversely proportional in their coagulation doses on different substrates, with Bm2 having a very low dose for plasma and a higher one for fibrinogen, while Bm4 having a higher dose for plasma and a lower one for fibrinogen. These differences can be explained by the fact that, while using plasma from any source, the venom toxins work in synergy to act in different steps of the blood coagulation cascade. On the other hand, using fibrinogen, the toxins act only in the last step of the coagulation mechanism, which involves consumption of the fibrinogen available in the circulation and formation of fibrin clot by action of the thrombin-like enzymes [89]. Therefore, we could assume that some individuals have proteases with thrombin-like characteristics, while others act in other points of the coagulation cascade.

For the next assays, we decided to use only the Bm2 and Bm4 individuals, the same ones used in the QTOF-nanoESI, so we could focus at the individual variability. If instead of that we decided to work with a pool of two pre-defined groups, these informations could be lost. Moreover, since we here prioritize to use the least amount of live animal for *in vivo* assays, test the LD50 and MHD of all of our thirteen individuals would be impracticable.

#### Minimum hemorrhagic dose

**Fig 9:**
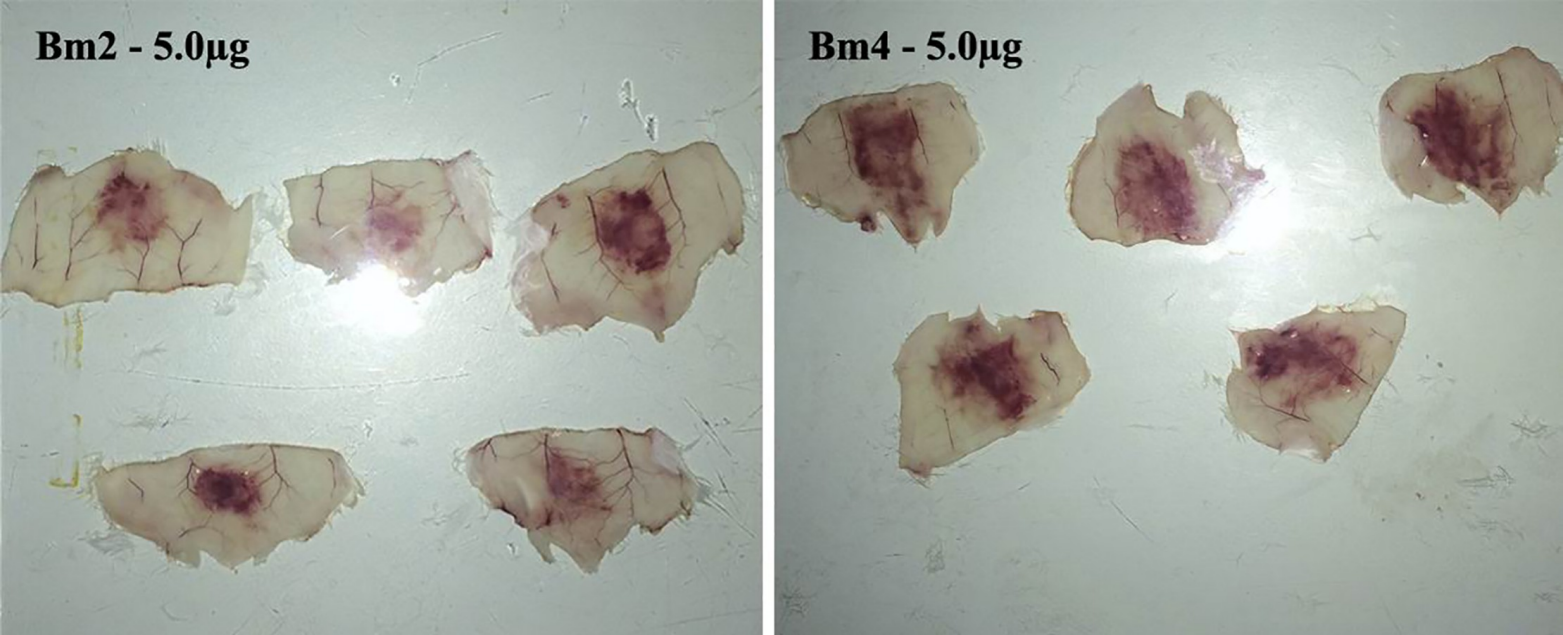
Hemorrhagic activity of two individual venoms of *B*. *moojeni* snakes. The hemorrhagic halos were the result of the intradermal injection of 5 μg of *B*. *moojeni* venom belonging to specimens Bm2 (A) and Bm4 (B).

The hemorrhagic activity of two individual venoms, Bm2 and Bm4, was evaluated by determination of MHD. Interestingly, Bm2 venom showed a MHD of 3.21 ±0.63 μg, whereas Bm4 venom presented a MHD approximately 52% lower (1.55 ±0.35μg). Fig 9 depicts the hemorrhagic halo induced by injection of 5 μg of venom.

#### Lethal dose _50_

**Fig 10:**
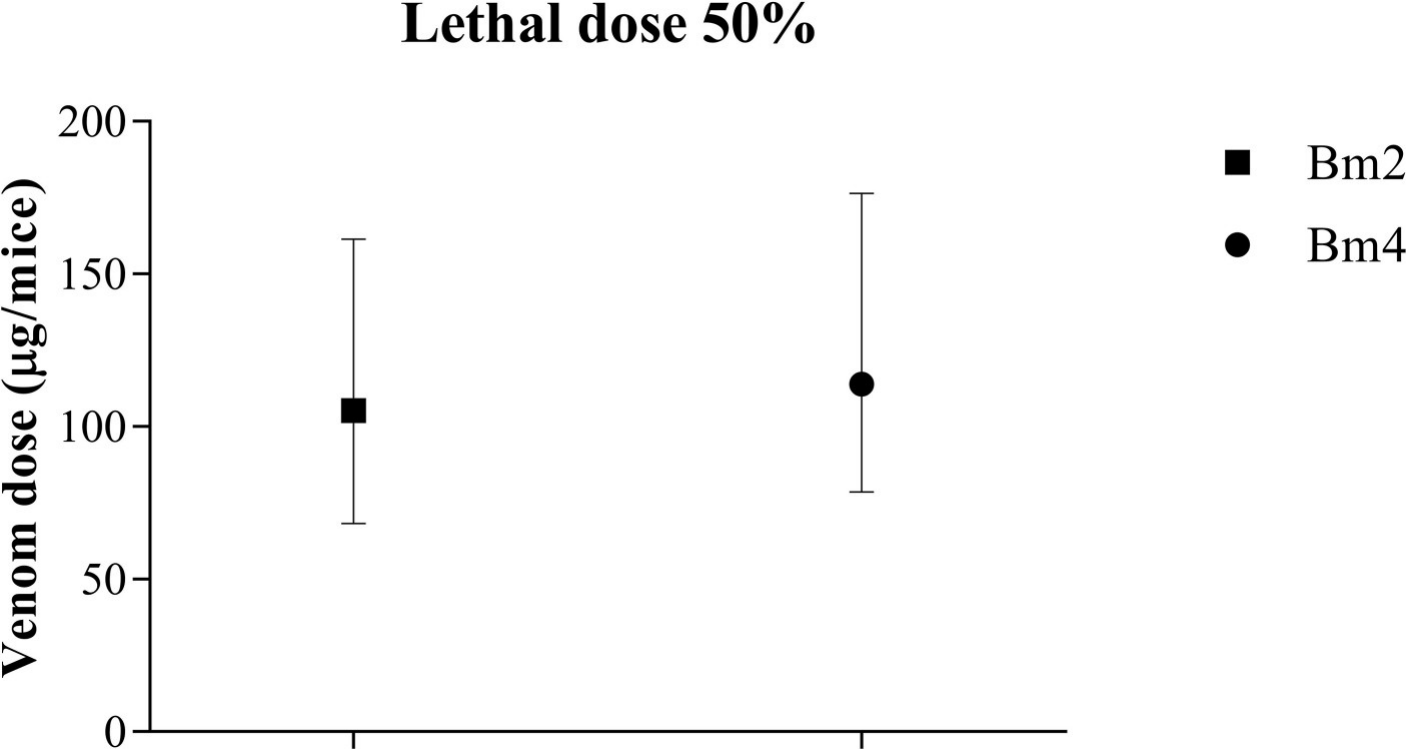
Lethal dose (LD_50_) of two individual venoms of *B*. *moojeni* snakes. The LD_50_ was determined by the intraperitoneal injection of different doses of *B*. *moojeni* venom belonging to the Bm2 and Bm4 individuals. Median lethal dose (LD_50_) is defined as the dose responsible for the death of 50% of the population analyzed.

The median lethal dose was determined using the same two venom samples used for determination of MHD, Bm2 and Bm4. During the experiment, 12 individuals from group Bm2 and 11 from Bm4 died. Both venoms showed comparable LD_50_ values (105 and 113 μg/animal, respectively), with no significant differences considering the lower and upper confidence limits of 95% (Fig 10).

LD_50_ is a standardize assay utilized to ensure the quality of the material during the preparation of venom batches utilized to produce the anti-venom serum [90] and is widely utilized in works that compare and characterize snake venoms, both from same and different species [42,91,92]. Comparison of LD_50_ and MHD between the Bm2 and Bm4 individuals indicated that venom with lower hemorrhagic dose (Bm4) has a tendency to a higher lethal dose. In contrast, venom with a tendency to a lower lethal dose presents increased hemorrhagic dose (Bm2). Even if no significant difference is found in the LD_50_, there is a tendency for this correlation, which has been previously documented. In 2012, Massey [16] conducted a study addressing the variability and severity of envenoming by snakes of the species *Crotalus scutulatus scutulatus* collected in counties in the states of Arizona and New Mexico, USA. The authors analyzed venoms of rattlesnakes from different locations and found that venom lethality decreased from central to southeastern regions. In addition, with this shift, the content of SVMP in those venoms also changed, with individuals with higher concentration of this compound showing lower lethality and individuals with lower contents showing a higher lethality.

It is also curious to note that both individuals in the present study showed an inverse result for the *in vitro* coagulation with fibrinogen and plasma. Individual Bm2, which was less hemorrhagic *in vivo*, presented faster coagulation time. Moreover, individual Bm4 showed higher hemorrhagic activity and poor coagulation capacity with plasma. This phenomenon can be explained by the multiple interactions and synergy that venom toxins have in animals during *in vivo* assays that cannot be fully reproduced *in vitro*. In addition to the consumption of fibrinogen in the organism by thrombin-like venom enzymes, they also cause bleeding in the injured individual.

Considering the results obtained by mass spectrometry, it can be observed that both venoms had a similar LD_50_ doses, but distinct DMH results. The venom that showed a slightly effective LD_50_, Bm2, had a lower relative abundance of SVMP compared with the Bm4 venom. Furthermore, as SVMP is closely related to hemostatic disorders such as bleeding, venoms with a higher concentration of these proteins present a more potent hemorrhagic activity. The variation in the properties of hemorrhagic and lethal activities of venoms found in *B*. *moojeni* has been confirmed by other studies [6,78,93], and it occurs within other snake genera, and even in other snakes of the *Bothrops* genus [94]. *B*. *jararaca*, for example, also presents this variation between venoms of adult and juvenile individuals [95]. This change is due to the hemorrhagic effect caused by these venoms—a metalloproteinase P-III type characteristic, which is more abundant in these individuals in their adulthood. SVMP content variation was also observed in this study, in which a higher amount of SVMP was found in the venom of Bm4, thus making it more hemorrhagic than Bm2.

## Concluding remarks

Unveiling snake venom profiles and their biological activities contributes to the elucidation of their complex envenomation mechanism and assists with future research. This study showed individual variability between the venoms of *B*. *moojeni* snake specimens. Moreover, these findings reinforce the diversity of venom phenotypes that can occur intraspecifically in many snake genera. It is also important to emphasize that putting all results together can contribute for the better understanding of the variability that has been proved to exist in *B*. *moojeni* and many other species. These studies also work for a greater good, when this type of analyses can contribute to other studies in the venomics and proteomics area, assisting with elucidating some of current proteomic questions. Therefore, it is in our best interest to contribute to the scientific development of this area, as a community, which tends to grow over time.

## Supporting information

S1 Raw Images Original images used in this work without any digital correction. (PDF)

S1 Fig Necropsy records of seven individuals of *B. moojeni* that showed signs of envenomation after being bitted by other individual of this species. Highlighted sessions of these files show a detailed analyses of different individuals with different symptoms.
1 –Two punctures in the right cranial region;
2 –Presence of blood in both the trachea and cranial region of the lungs;
3 –Thickened pericardium and hemorrhage in the aorta region.
4 –Macroscopic: hemorrhage;
5 –Blood vessels engorged and general hypovolemia in the byte region (cranial and 1/3 medium);
6 –Congested esophagus around the byte site;
7 –Congested testicles. Left testicle atrophied;
8 –Presence of blood cloth and hemorrhage in the 1/3 medial region. Reddish musculature around the byte site;
9 –Thickened pericardium with hemorrhage through the aorta;
10 –Congested testicles;
10 –Congested testicle;
11 –Blood vessels engorged at the superior region;
12 –Congested lungs. Hemorrhage at the cranial portion of the lungs;
13 –Engorged blood vessels at the byte site. Hypovolemia;
14 –Congested testicles;
15 –Engorged blood vessels at the medium region of the body (possible site of the byte);
16 –Yellowish liver. Presence of blood cloths in the caudal region;
17 –Congested testicles.
(TIF)

S2 Fig SDS-Page scan of the protein bands excised and submitted to *in–gel* trypsin digestion. Samples from Bm1 to Bm7 belongs to *B*. *moojeni* plasma sample, while Bj8 to Bj13 it is from *B*. *jararaca*.
(PDF)

S1 Table Proteins identified in individual venom from *B. moojeni* snakes individuals Bm2 and Bm4. Quantitative data were obtained through label-free quantification by high resolution LC-MS/MS considering the averaged intensities of the three most intense peptides of each identified protein.
(XLSX)

S2 Table List of all plasma protein from *B. moojeni* identified by mass spectrometry, listed by: sample number, protein accession code and toxin class. Quantitative data were obtained through label-free quantification by high resolution LC-MS. Sample numbers can be verified in S2 Fig.
(XLSX)
