# Supplementary figures and images for: Comparative compositional and functional analyses of Bothrops moojeni specimens reveal several individual variations
Source: PLoS One. 2019 Sep 12;14(9):e0222206. doi: 10.1371/journal.pone.0222206 (PMC6742229; doi:10.1371/journal.pone.0222206)

**Fig 2.**

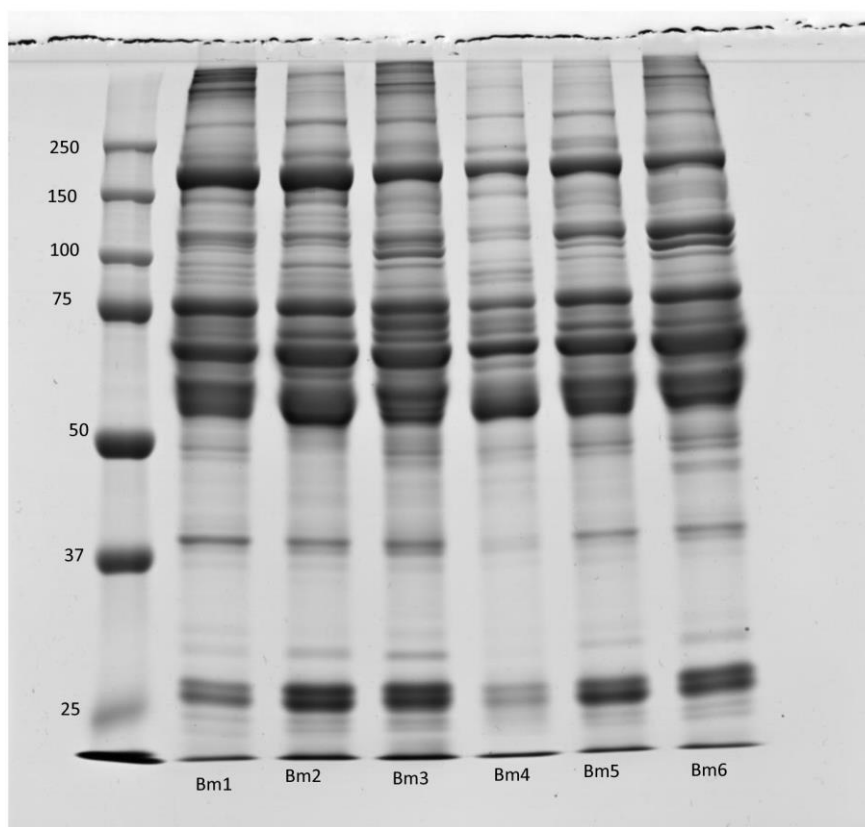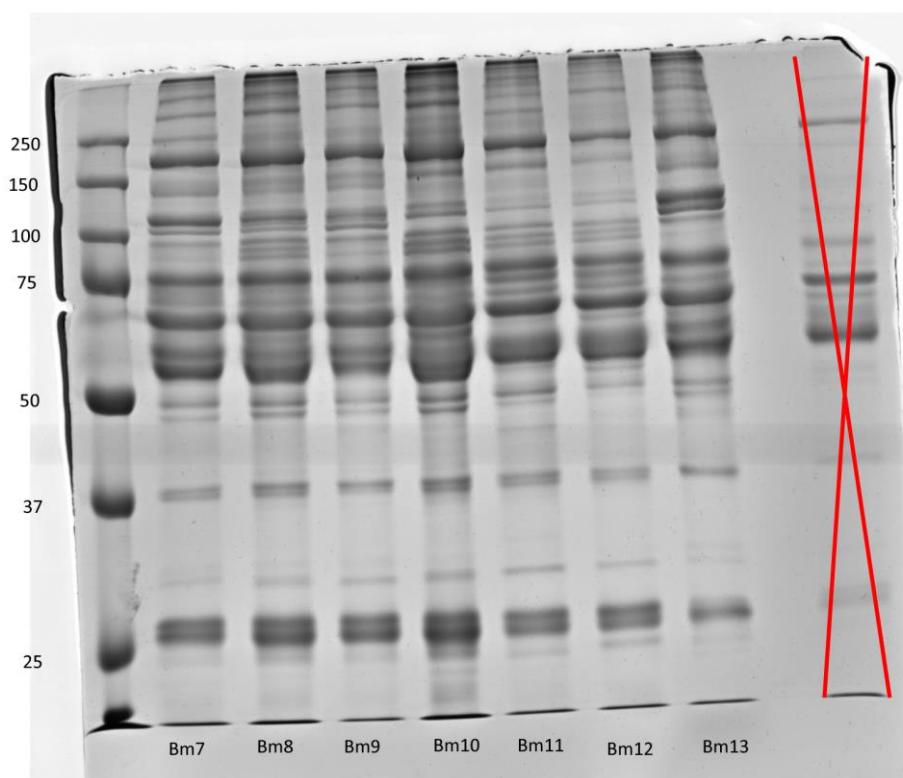

Fig 3.

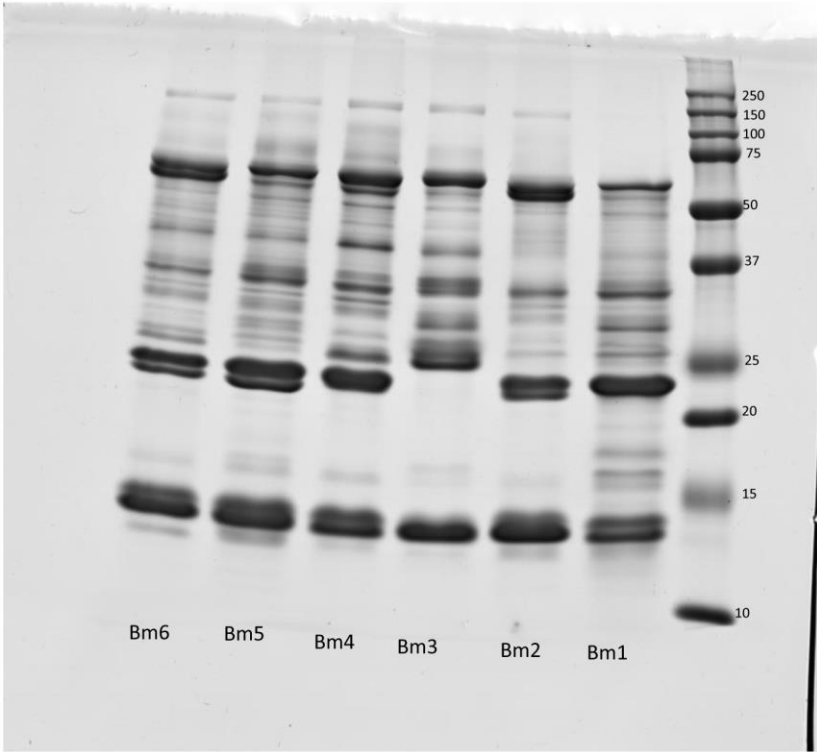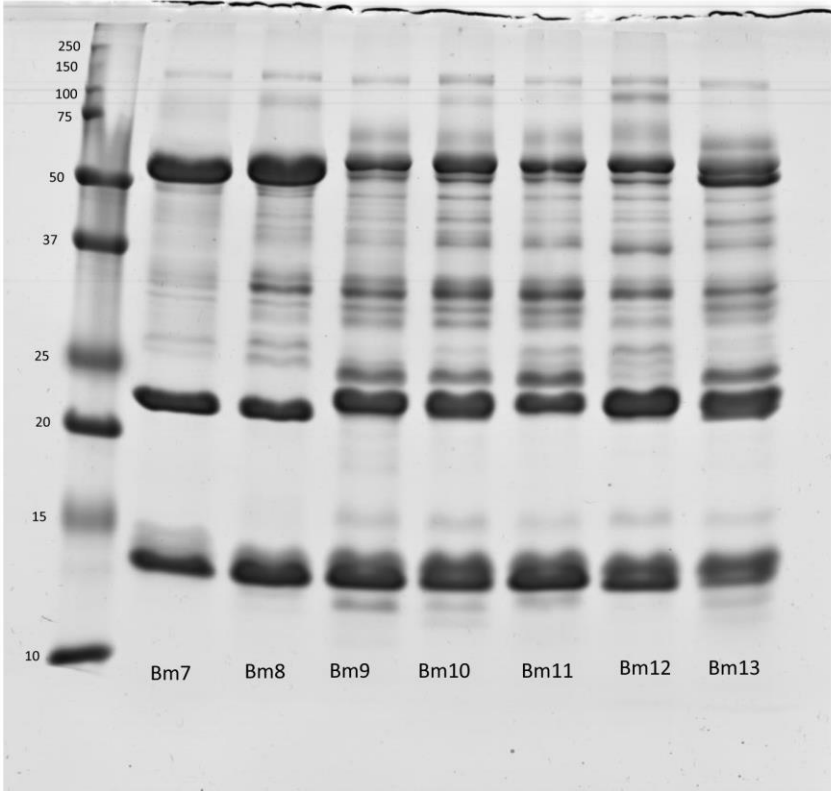

**Fig 4.**

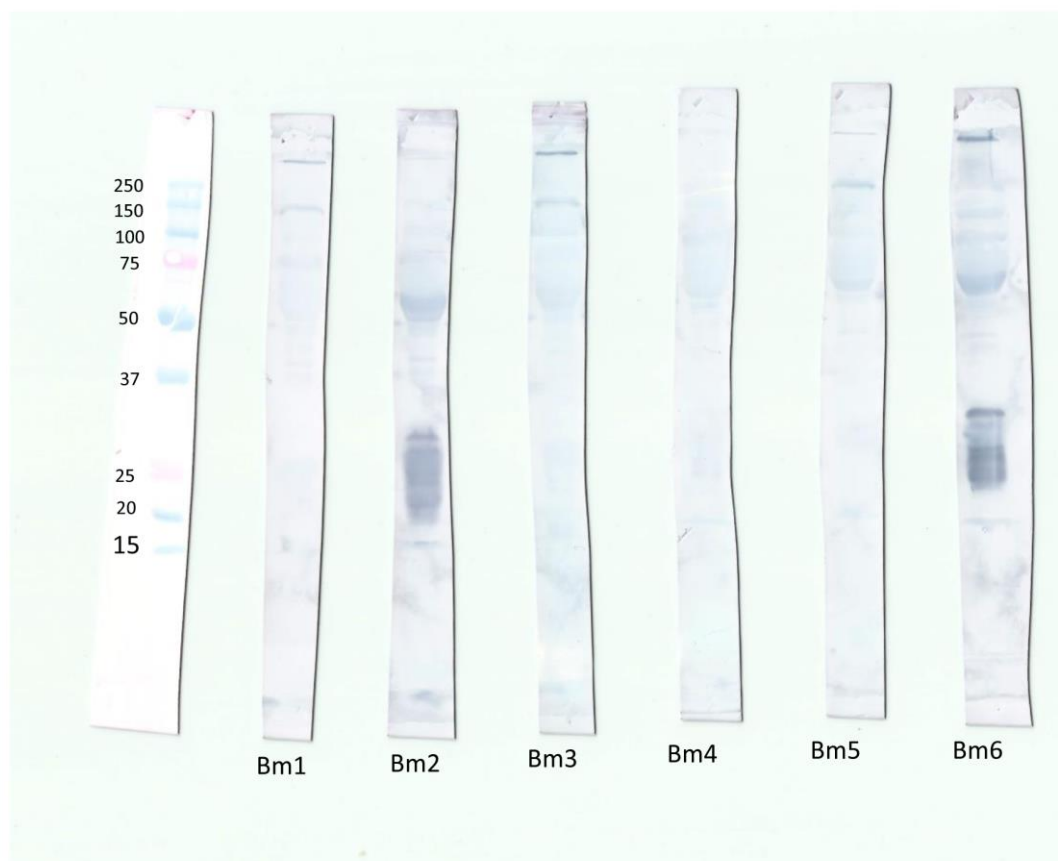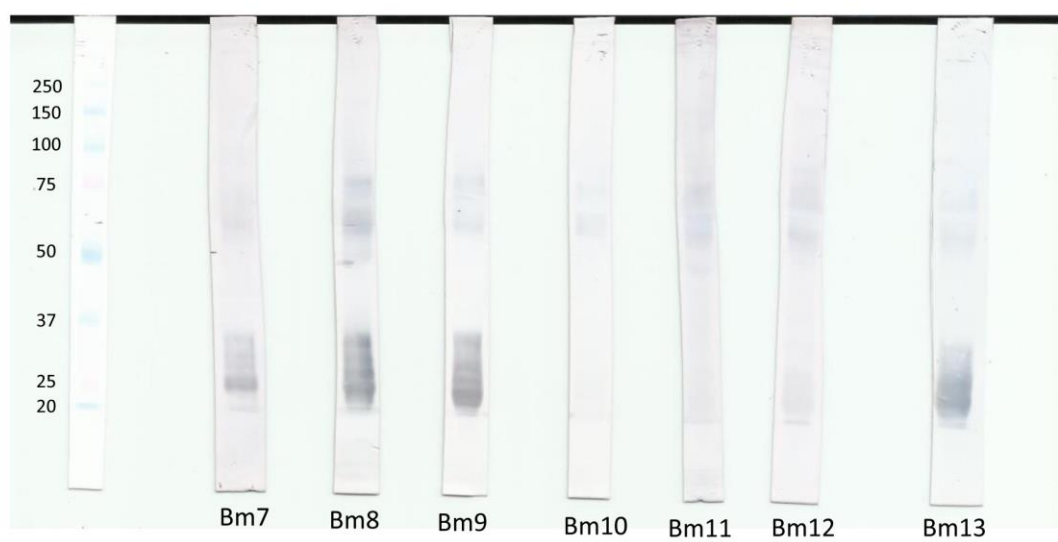

**Fig 5.**

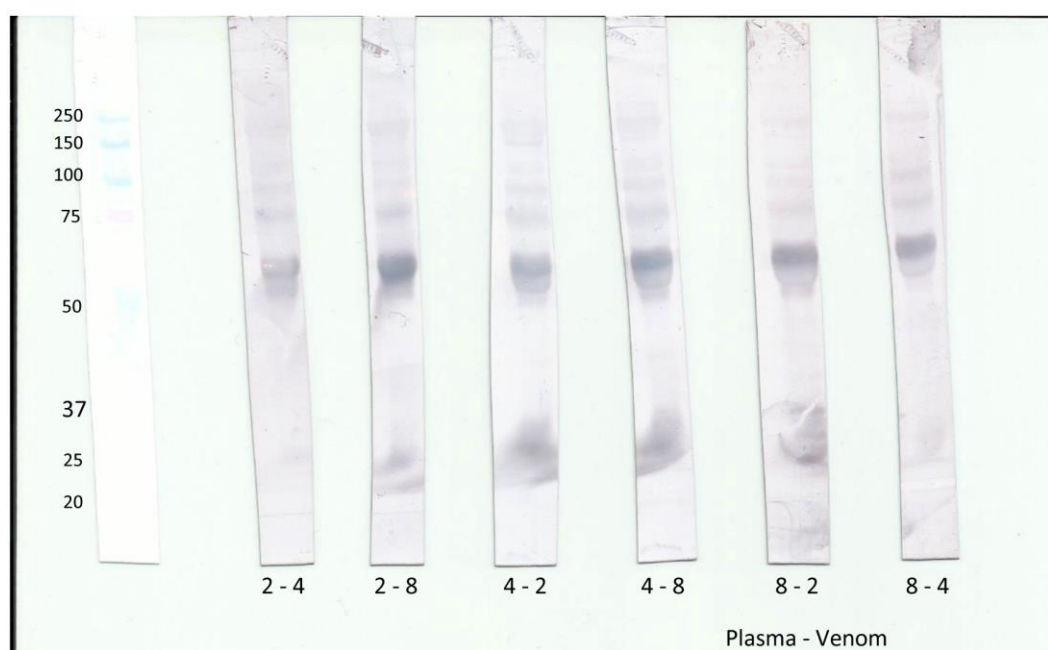

**Supplementary figure 1.**

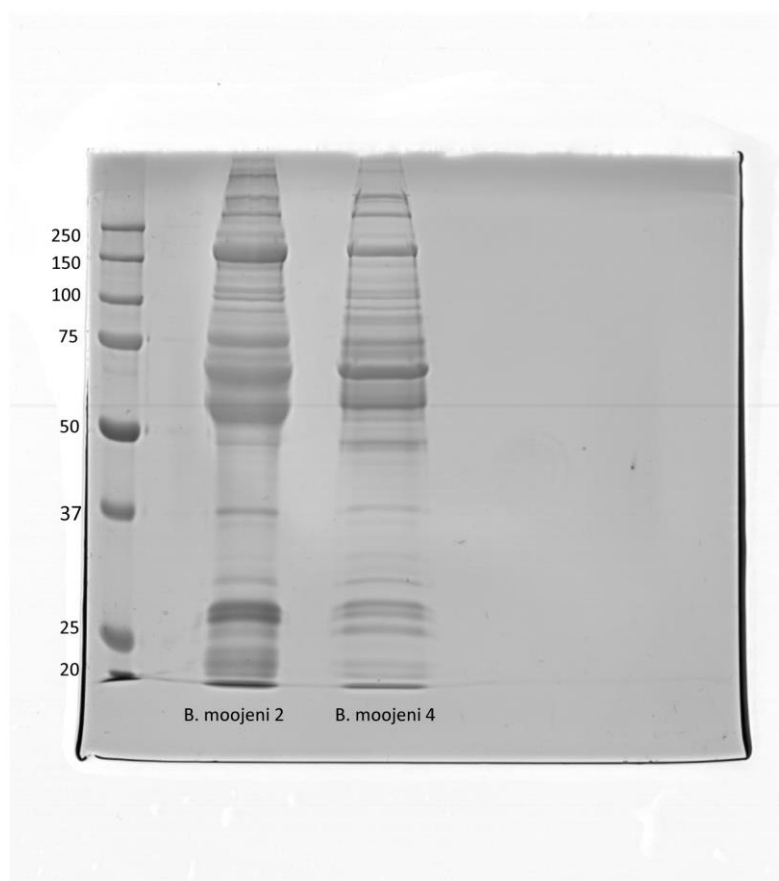

Supplement: S1 Raw Images — (PDF) [file pone.0222206.s001.pdf]

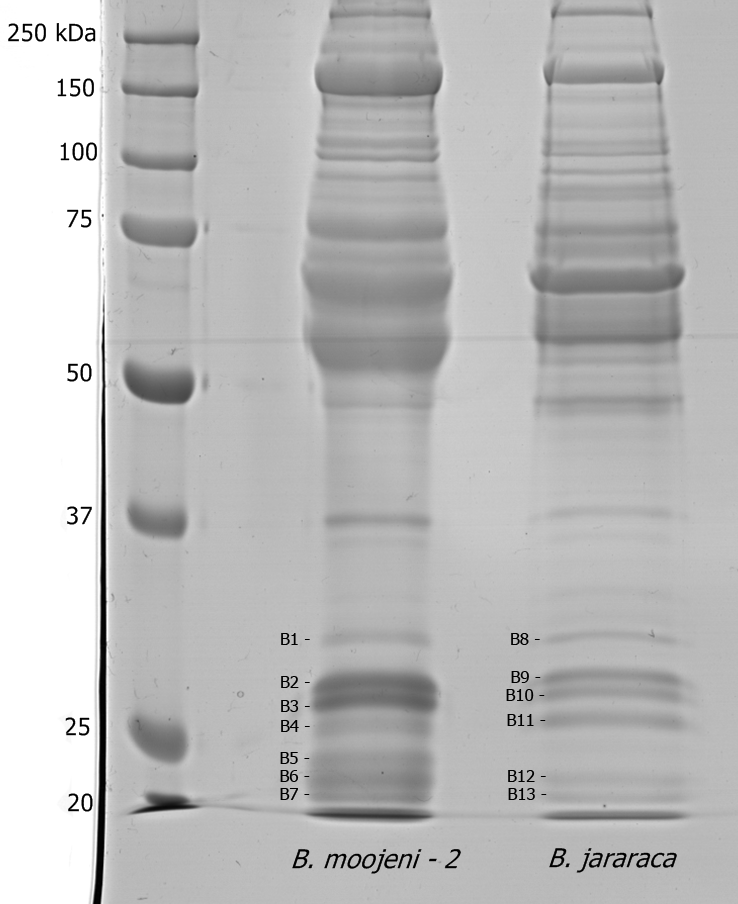

Supplement: S1 Fig — Highlighted sessions of these files show a detailed analyses of different individuals with different symptoms. 1 –Two punctures in the right cranial region; 2 –Presence of blood in both the trachea and cranial region of the lungs; 3 –Thickened pericardium and hemorrhage in the aorta region. 4 –Macroscopic: hemorrhage; 5 –Blood vessels engorged and general hypovolemia in the byte region (cranial and 1/3 medium); 6 –Congested esophagus around the byte site; 7 –Congested testicles. Left testicle atrophied; 8 –Presence of blood cloth and hemorrhage in the 1/3 medial region. Reddish musculature around the byte site; 9 –Thickened pericardium with hemorrhage through the aorta; 10 –Congested testicles; 10 –Congested testicle; 11 –Blood vessels engorged at the superior region; 12 –Congested lungs. Hemorrhage at the cranial portion of the lungs; 13 –Engorged blood vessels at the byte site. Hypovolemia; 14 –Congested testicles; 15 –Engorged blood vessels at the medium region of the body (possible site of the byte); 16 –Yellowish liver. Presence of blood cloths in the caudal region; 17 –Congested testicles. (TIF) [file pone.0222206.s002.tif]
