# Supplementary material for: Comparative compositional and functional analyses of Bothrops moojeni specimens reveal several individual variations
Source: PLoS One. 2019 Sep 12;14(9):e0222206. doi: 10.1371/journal.pone.0222206 (PMC6742229; doi:10.1371/journal.pone.0222206)
Supplement: S2 Fig — Samples from Bm1 to Bm7 belongs to B. moojeni plasma sample, while Bj8 to Bj13 it is from B. jararaca. (PDF) [file pone.0222206.s003.pdf]

FN: 2342

FICHA DE NECROPSIA

**Código do animal:** Bm0807	**Data de óbito:** 19/8/11	**Data de necropsia:** 19/8/11
**Origem:** S. 4	**Comprimento (cm):** 28,5-102,5	**Peso (g):** 200g **Sexo:** ♂

Histórico do animal:

Estava com uma fêmea na sala 7

ESTADO GERAL:
( )bom (X) regular ( ) caquético 1

PELE: (X) ndn ( ) em ecdise ( )disecdise
(X) outros: 2 perfurações de dente, na região frontal lado direito

MUCOSA ORAL: ( ) ndn
Coloração: ( ) normocorada ( ) hipocorada (X) congesta ( ) cianótica ( ) hemorrágica discretamente
Estomatite: ( ) discreta ( ) moderada ( ) severa
Secreção: ( )catarral ( )caseosa Quantidade: ( ) pouca ( ) muita

TRAQUÉIA: ( ) ndn
Secreção: ( ) mucosa ( ) serosa ( )purulenta ( ) caseosa
Parasitos:
Outros: presença de sangue

2

PULMÃO: (X) ndn
Coloração: ( ) normocorado ( )hipocorado ( ) congesto ( ) hemorrágico ( ) cianótico
Edema: ( ) discreto ( ) moderado ( ) severo
Secreção: ( ) purulenta ( ) caseosa ( ) placas caseosas ( ) nódulos caseosos
Parasitos:
Outros: região cranial congesto e c/hemorragia

SACO AÉREO: (X) ndn
Secreção: ( ) serosa ( ) mucosa ( ) purulenta ( ) caseosa
Parasitos:
Outros:

TIREÓIDE: ( ) ndn ( ) atrofiada ( ) aumentada ( ) hipocorada ( ) congesta
Outros:

CORAÇÃO: ( ) ndn
Pericárdio: ( ) ndn (X)espessado (X) hidropericárdio ( ) hemopericárdio ( ) parasitos
Miocárdio: (X) ndn ( ) atrofiado ( ) hipertrofiado ( ) congesto ( ) hipocorado
( ) nódulos caseosos ( )áreas de infarto ( ) outros:
Valva: ( ) ndn ( )espessadas ( ) outros:
Parasitos:

VASOS SANGUÍNEOS: (X) ndn ( ) engurgitados ( ) hipovolemia ( ) nódulos no endotélio
Outros:

FÍGADO: ( ) ndn
Coloração: ( ) vinhácea (X) amarronzado ( ) amarelado ( ) congesto ( ) aparência noz moscada
Volume: ( ) atrofiado ( ) hepatomegalia / Consistência: (X) firme ( ) friável
Nódulos caseosos: ( ) focais ( ) difusos / Áreas de infarto: ( ) focais ( ) difusos
Outros:

ESÔFAGO: (X) ndn
Mucosa: ( ) ndn (X) congesta discretamente ( ) espessada ( ) hemorrágica ( ) ictérica
Secreção: ( ) serosa ( ) mucosa ( ) catarral ( ) purulenta ( ) caseosa ( ) enegrecida ( ) fétida
Outros:

ESTÔMAGO: (X) ndn
Mucosa: ( ) ndn ( ) congesta ( ) espessada ( ) hemorrágica ( ) ictérica
Secreção: ( ) serosa ( ) mucosa ( ) catarral ( ) purulenta ( ) caseosa ( ) enegrecida ( ) fétida
Outros:

INTESTINO: ( ) ndn
Mucosa: ( ) ndn (X) congesta ( ) espessada ( ) hemorrágica ( ) ictérica
Secreção: ( ) serosa ( ) mucosa ( ) catarral (X) purulenta ( ) caseosa ( ) enegrecida ( ) fétida
Outros: porção final do intestino hemorrágica c/ secreção purulenta

PÂNCREAS: (X) ndn
Coloração: ( ) normocorado ( ) hipocorado ( ) congesto ( ) hemorrágico
Volume: ( ) atrofiado ( ) hipertrofiado / Consistência: ( ) firme ( ) friável
Nódulos caseosos: ( ) focais ( ) difusos / Áreas de infarto: ( ) focais ( ) difusos
Outros:

BAÇO: (X) ndn
Coloração: ( ) normocorado ( ) hipocorado ( ) congesto ( ) hemorrágico
Volume: ( ) atrofiado ( ) hipertrofiado / Consistência: ( ) firme ( ) friável
Nódulos caseosos: ( ) focais ( ) difusos / Áreas de infarto: ( ) focais ( ) difusos
Outros:

VESÍCULA BILIAR: ( ) ndn (X) cheia ( ) parede espessada ( ) cálculos ( ) parasitos
Outros: cor: amarelo amarronzado

TESTÍCULOS: (X) ndn
Coloração: ( ) normocorado ( ) hipocorado ( ) congesto ( ) hemorrágico
Volume: ( ) atrofiado ( ) hipertrofiado / Consistência: ( ) firme ( ) friável
Nódulos caseosos: ( ) focais ( ) difusos / Áreas de infarto: ( ) focais ( ) difusos
Outros:

OVÁRIOS: ( ) ndn
Coloração: ( ) normocorado ( ) hipocorado ( ) congesto ( ) hemorrágico
Folículos ovarianos: ( ) ausentes / No estágio: ( ) I ( ) II ( ) III ( ) IV ( ) V ( ) VI
Corpos lúteos: ( ) ausentes ( ) presentes : quantidade
Ovidutos: ( ) ndn ( ) parede espessada ( ) congesto ( ) recém-parida ( ) placas caseosas

RINS: (X) ndn
Coloração: ( ) normocorado ( ) hipocorado ( ) congesto ( ) hemorrágico
Volume: ( ) atrofiado ( ) hipertrofiado / Consistência: ( ) firme ( ) friável
Nódulos caseosos: ( ) focais ( ) difusos / Áreas de infarto: ( ) focais ( ) difusos
Outros:

ADRENAIS: (X) ndn ( ) hipocoradas ( ) congestas ( ) hemorrágicas
Outros:

GORDURA CELOMÁTICA: ( ) ndn (X) boa quantidade ( ) escassa ( ) quantidade regular ( ) excesso ( ) aavermelhada ( ) petéquias ( ) nódulos caseosos
Outros:

CELOMA: (X) ndn ( ) presença de transudato ( ) presença de exsudato ( ) parasitos

MUSCULATURA: ( ) ndn região escamosa lado ~~esq.~~ direito c/ hematoma (na altura do [illegible]

SISTEMA ÓSSEO: (X) ndn

DIAGNÓSTICO MACROSCÓPICO:

musculatura: lesão na musculatura

□

963000000250206 **FICHA DE NECROPSIA** FN: 2172

**Código do animal:** B. moojeni	**Data de óbito:** 12/11/09	**Data de necropsia:** 17.11.09
**Origem:** Ser. produção	**Comprimento (cm):** 85-98,5	**Peso (g):** 260g **Sexo:** ♂

Histórico do animal:

Levou picada de outra moojeni

ESTADO GERAL:
(X) bom ( ) regular ( ) caquético

PELE: (X) ndn ( ) em ecdise ( ) disecdise
( ) outros:

MUCOSA ORAL: ( ) ndn
Coloração: ( ) normocorada ( ) hipocorada ( ) congesta ( ) cianótica (X) hemorrágica
Estomatite: ( ) discreta ( ) moderada ( ) severa
Secreção: ( ) catarral ( ) caseosa Quantidade: ( ) pouca ( ) muita

TRAQUÉIA: (X) ndn
Secreção: ( ) mucosa ( ) serosa ( ) purulenta ( ) caseosa
Parasitos:
Outros:

PULMÃO: (X) ndn
Coloração: ( ) normocorado ( ) hipocorado ( ) congesto ( ) hemorrágico ( ) cianótico
Edema: ( ) discreto ( ) moderado ( ) severo
Secreção: ( ) purulenta ( ) caseosa ( ) placas caseosas ( ) nódulos caseosos
Parasitos:
Outros:

SACO AÉREO: (X) ndn
Secreção: ( ) serosa ( ) mucosa ( ) purulenta ( ) caseosa
Parasitos:
Outros:

TIREÓIDE: (X) ndn ( ) atrofiada ( ) aumentada ( ) hipocorada ( ) congesta
Outros:

CORAÇÃO: ( ) ndn
Pericárdio: ( ) ndn (X) espessado ( ) hidropericárdio ( ) hemopericárdio ( ) parasitos
3 Miocárdio: ( ) ndn ( ) atrofiado ( ) hipertrofiado ( ) congesto (X) hipocorado
( ) nódulos caseosos ( ) áreas de infarto ( ) outros:
Valva: ( ) ndn ( ) espessadas ( ) outros:
Parasitos: região da aorta c/ hemorragia

VASOS SANGUÍNEOS: ( ) ndn ( ) engurgitados (X) hipovolemia ( ) nódulos no endotélio
Outros:

FÍGADO: (X) ndn
Coloração: ( ) vinhácea ( ) amarronzado ( ) amarelado ( ) congesto ( ) aparência noz moscada
Volume: ( ) atrofiado ( ) hepatomegalia / Consistência: ( ) firme ( ) friável
Nódulos caseosos: ( ) focais ( ) difusos / Áreas de infarto: ( ) focais ( ) difusos
Outros:

ESÔFAGO: (X) ndn
Mucosa: ( ) ndn ( ) congesta ( ) espessada ( ) hemorrágica ( ) ictérica
Secreção: ( ) serosa ( ) mucosa ( ) catarral ( ) purulenta ( ) caseosa ( ) enegrecida ( ) fétida
Outros:

ESTÔMAGO: (X) ndn
Mucosa: ( ) ndn ( ) congesta ( ) espessada ( ) hemorrágica ( ) ictérica
Secreção: ( ) serosa ( ) mucosa ( ) catarral ( ) purulenta ( ) caseosa ( ) enegrecida ( ) fétida
Outros:

INTESTINO: (X) ndn
Mucosa: ( ) ndn ( ) congesta ( ) espessada ( ) hemorrágica ( ) ictérica
Secreção: ( ) serosa ( ) mucosa ( ) catarral ( ) purulenta ( ) caseosa ( ) enegrecida ( ) fétida
Outros:

PÂNCREAS: (X) ndn
Coloração: ( ) normocorado ( ) hipocorado ( ) congesto ( ) hemorrágico
Volume: ( ) atrofiado ( ) hipertrofiado / Consistência: ( ) firme ( ) friável
Nódulos caseosos: ( ) focais ( ) difusos / Áreas de infarto: ( ) focais ( ) difusos
Outros:

BAÇO: (X) ndn
Coloração: ( ) normocorado ( ) hipocorado ( ) congesto ( ) hemorrágico
Volume: ( ) atrofiado ( ) hipertrofiado / Consistência: ( ) firme ( ) friável
Nódulos caseosos: ( ) focais ( ) difusos / Áreas de infarto: ( ) focais ( ) difusos
Outros:

VESÍCULA BILIAR: (X) ndn ( ) cheia ( ) parede espessada ( ) cálculos ( ) parasitos
Outros:

TESTÍCULOS: (X) ndn
Coloração: ( ) normocorado ( ) hipocorado ( ) congesto ( ) hemorrágico
Volume: ( ) atrofiado ( ) hipertrofiado / Consistência: ( ) firme ( ) friável
Nódulos caseosos: ( ) focais ( ) difusos / Áreas de infarto: ( ) focais ( ) difusos
Outros:

OVÁRIOS: ( ) ndn
Coloração: ( ) normocorado ( ) hipocorado ( ) congesto ( ) hemorrágico
Folículos ovarianos: ( ) ausentes / No estágio: ( ) I ( ) II ( ) III ( ) IV ( ) V ( ) VI
Corpos lúteos: ( ) ausentes ( ) presentes : quantidade
Ovidutos: ( ) ndn ( ) parede espessada ( ) congesto ( ) recém-parida ( ) placas caseosas

RINS: ( ) ndn
Coloração: ( ) normocorado (X) hipocorado ( ) congesto ( ) hemorrágico
Volume: ( ) atrofiado ( ) hipertrofiado / Consistência: ( ) firme ( ) friável
Nódulos caseosos: ( ) focais ( ) difusos / Áreas de infarto: ( ) focais ( ) difusos
Outros:

ADRENAIS: (X) ndn ( ) hipocoradas ( ) congestas ( ) hemorrágicas
Outros:

GORDURA CELOMÁTICA: ( ) ndn (X) boa quantidade ( ) escassa ( ) quantidade regular ( ) excesso ( ) aavermelhada ( ) petéquias ( ) nódulos caseosos
Outros:

CELOMA: (X) ndn ( ) presença de transudato ( ) presença de exsudato ( ) parasitos

MUSCULATURA: (X) ndn

SISTEMA ÓSSEO: (X) ndn

DIAGNÓSTICO MACROSCÓPICO:
hemorragia

963000000277244 **FICHA DE NECROPSIA** | FN: 2176

**Código do animal:** B. moojeni	**Data de óbito:** 12/11/09	**Data de necropsia:** 17/11/09
**Origem:** serp produção	**Comprimento (cm):** ~~[illegible]~~ 96,5-112	**Peso (g):** 280g	**Sexo:** ♂

Histórico do animal:

picado por outra moojeni

ESTADO GERAL:
(X) bom ( ) regular ( ) caquético

PELE: (X) ndn ( ) em ecdise ( ) disecdise
( ) outros:

MUCOSA ORAL: ( ) ndn
Coloração: ( ) normocorada ( ) hipocorada (X) congesta ( ) cianótica ( ) hemorrágica
Estomatite: ( ) discreta ( ) moderada ( ) severa
Secreção: (X) catarral ( ) caseosa Quantidade: (X) pouca ( ) muita

TRAQUÉIA: (X) ndn
Secreção: ( ) mucosa ( ) serosa ( ) purulenta ( ) caseosa
Parasitos:
Outros:

PULMÃO: ( ) ndn
Coloração: ( ) normocorado (X) hipocorado ( ) congesto ( ) hemorrágico ( ) cianótico
Edema: ( ) discreto (X) moderado ( ) severo
Secreção: ( ) purulenta ( ) caseosa ( ) placas caseosas ( ) nódulos caseosos
Parasitos:
Outros:

SACO AÉREO: (X) ndn
Secreção: ( ) serosa ( ) mucosa ( ) purulenta ( ) caseosa
Parasitos:
Outros:

TIREÓIDE: (X) ndn ( ) atrofiada ( ) aumentada ( ) hipocorada ( ) congesta
Outros:

CORAÇÃO: ( ) ndn
Pericárdio: ( ) ndn (X) espessado ( ) hidropericárdio ( ) hemopericárdio ( ) parasitos
Miocárdio: ( ) ndn ( ) atrofiado ( ) hipertrofiado ( ) congesto (X) hipocorado
( ) nódulos caseosos ( ) áreas de infarto ( ) outros:
Valva: ( ) ndn ( ) espessadas ( ) outros:
Parasitos:

5 **VASOS SANGUÍNEOS:** ( ) ndn (X) engurgitados (X) hipovolemia ( ) nódulos no endotélio → no geral
Outros: na região da picada (cranial e no 1/3 médio)

FÍGADO: ( ) ndn
Coloração: ( ) vinhácea (X) amarronzado ( ) amarelado ( ) congesto ( ) aparência noz moscada
Volume: ( ) atrofiado ( ) hepatomegalia / Consistência: ( ) firme ( ) friável
Nódulos caseosos: ( ) focais ( ) difusos / Áreas de infarto: ( ) focais (X) difusos
Outros:

6 **ESÔFAGO:** ~~( )~~ ndn → perto do local da picada
Mucosa: ( ) ndn (X) congesta ( ) espessada ( ) hemorrágica ( ) ictérica
Secreção: ( ) serosa ( ) mucosa ( ) catarral ( ) purulenta ( ) caseosa ( ) enegrecida ( ) fétida
Outros:

ESTÔMAGO: (X) ndn
Mucosa: ( ) ndn ( ) congesta ( ) espessada ( ) hemorrágica ( ) ictérica
Secreção: ( ) serosa ( ) mucosa ( ) catarral ( ) purulenta ( ) caseosa ( ) enegrecida ( ) fétida
Outros:

INTESTINO: (X) ndn
Mucosa: ( ) ndn ( ) congesta ( ) espessada ( ) hemorrágica ( ) ictérica
Secreção: ( ) serosa ( ) mucosa ( ) catarral ( ) purulenta ( ) caseosa ( ) enegrecida ( ) fétida
Outros:

PÂNCREAS: ( ) ndn
Coloração: ( ) normocorado ( ) hipocorado ( ) congesto ( ) hemorrágico
Volume: ( ) atrofiado ( ) hipertrofiado / Consistência: ( ) firme ( ) friável
Nódulos caseosos: ( ) focais ( ) difusos / Áreas de infarto: ( ) focais ( ) difusos
Outros:
autólise

BAÇO: ( ) ndn
Coloração: ( ) normocorado ( ) hipocorado ( ) congesto ( ) hemorrágico
Volume: ( ) atrofiado ( ) hipertrofiado / Consistência: ( ) firme ( ) friável
Nódulos caseosos: ( ) focais ( ) difusos / Áreas de infarto: ( ) focais ( ) difusos
Outros:
autólise

VESÍCULA BILIAR: ( ) ndn ( ) cheia ( ) parede espessada ( ) cálculos ( ) parasitos
Outros: vazia (um pouco)

TESTÍCULOS: ( ) ndn
Coloração: ( ) normocorado ( ) hipocorado (X) congesto ( ) hemorrágico
7 Volume: ( ) atrofiado ( ) hipertrofiado / Consistência: ( ) firme ( ) friável
Nódulos caseosos: ( ) focais ( ) difusos / Áreas de infarto: ( ) focais ( ) difusos
Outros: o testículo esquerdo atrofiado

OVÁRIOS: ( ) ndn
Coloração: ( ) normocorado ( ) hipocorado ( ) congesto ( ) hemorrágico
Folículos ovarianos: ( ) ausentes / No estágio: ( ) I ( ) II ( ) III ( ) IV ( ) V ( ) VI
Corpos lúteos: ( ) ausentes ( ) presentes / quantidade
Ovidutos: ( ) ndn ( ) parede espessada ( ) congesto ( ) recém-parida ( ) placas caseosas

RINS: ( ) ndn
→ aparência gelatinosa
Coloração: ( ) normocorado (X) hipocorado ( ) congesto ( ) hemorrágico
Volume: ( ) atrofiado ( ) hipertrofiado / Consistência: ( ) firme ( ) friável
Nódulos caseosos: ( ) focais ( ) difusos / Áreas de infarto: ( ) focais ( ) difusos
Outros:

ADRENAIS: (X) ndn ( ) hipocoradas ( ) congestas ( ) hemorrágicas
Outros:

GORDURA CELOMÁTICA: ( ) ndn (X) boa quantidade ( ) escassa ( ) quantidade regular ( ) excesso ( ) aavermelhada ( ) petéquias ( ) nódulos caseosos
Outros:

CELOMA: ( ) ndn ( ) presença de transudato ( ) presença de exsudato ( ) parasitos

MUSCULATURA: ( ) ndn musculatura avermelhada na área da picada

SISTEMA ÓSSEO: (X) ndn

8 **DIAGNÓSTICO MACROSCÓPICO:**

não evidente

→ presença de coágulo e hemorragia no 1/3 medial

963000000284479 **FICHA DE NECROPSIA**

FN: 2174

**Código do animal:** B.moojeni	**Data de óbito:** 12/11/09	**Data de necropsia:** 17/11/09
**Origem:** perf. prod.	**Comprimento (cm):** 88-108	**Peso (g):** 290 — **Sexo:** ♂

Histórico do animal:
Picado por outro animal.

ESTADO GERAL:
(X) bom ( ) regular ( ) caquético

PELE: (X) ndn ( ) em ecdise ( ) disecdise
( ) outros:

MUCOSA ORAL: (X) ndn
Coloração: ( ) normocorada ( ) hipocorada ( ) congesta ( ) cianótica ( ) hemorrágica
Estomatite: ( ) discreta ( ) moderada ( ) severa
Secreção: ( ) catarral ( ) caseosa Quantidade: ( ) pouca ( ) muita

TRAQUÉIA: (X) ndn
Secreção: ( ) mucosa ( ) serosa ( ) purulenta ( ) caseosa
Parasitos:
Outros:

PULMÃO: (X) ndn
Coloração: ( ) normocorado ( ) hipocorado ( ) congesto ( ) hemorrágico ( ) cianótico
Edema: ( ) discreto ( ) moderado ( ) severo
Secreção: ( ) purulenta ( ) caseosa ( ) placas caseosas ( ) nódulos caseosos
Parasitos:
Outros:

SACO AÉREO: (X) ndn
Secreção: ( ) serosa ( ) mucosa ( ) purulenta ( ) caseosa
Parasitos:
Outros:

TIREÓIDE: (X) ndn ( ) atrofiada ( ) aumentada ( ) hipocorada ( ) congesta
Outros:

CORAÇÃO: ( ) ndn
Pericárdio: ( ) ndn (X) espessado ( ) hidropericárdio ( ) hemopericárdio ( ) parasitos
9 Miocárdio: ( ) ndn ( ) atrofiado ( ) hipertrofiado ( ) congesto ( ) hipocorado
( ) nódulos caseosos ( ) áreas de infarto ( ) outros:
Valva: ( ) ndn ( ) espessadas ( ) outros:
Parasitos: hemorragia pela aorta

VASOS SANGUÍNEOS: ( ) ndn ( ) engurgitados (X) hipovolemia ( ) nódulos no endotélio
Outros:

FÍGADO: (X) ndn
Coloração: ( ) vinhácea (X) amarronzado ( ) amarelado ( ) congesto ( ) aparência noz moscada
Volume: ( ) atrofiado ( ) hepatomegalia / Consistência: ( ) firme ( ) friável
Nódulos caseosos: ( ) focais ( ) difusos / Áreas de infarto: ( ) focais ( ) difusos
Outros:

ESÔFAGO: (X) ndn
Mucosa: ( ) ndn ( ) congesta ( ) espessada ( ) hemorrágica ( ) ictérica
Secreção: ( ) serosa ( ) mucosa ( ) catarral ( ) purulenta ( ) caseosa ( ) enegrecida ( ) fétida
Outros:

ESTÔMAGO: (X) ndn
Mucosa: ( ) ndn ( ) congesta ( ) espessada ( ) hemorrágica ( ) ictérica
Secreção: ( ) serosa ( ) mucosa ( ) catarral ( ) purulenta ( ) caseosa ( ) enegrecida ( ) fétida
Outros:

INTESTINO: (X) ndn
Mucosa: ( ) ndn ( ) congesta ( ) espessada ( ) hemorrágica ( ) ictérica
Secreção: ( ) serosa ( ) mucosa ( ) catarral ( ) purulenta ( ) caseosa ( ) enegrecida ( ) fétida
Outros:

PÂNCREAS: (X) ndn
Coloração: ( ) normocorado ( ) hipocorado ( ) congesto ( ) hemorrágico
Volume: ( ) atrofiado ( ) hipertrofiado / Consistência: ( ) firme ( ) friável
Nódulos caseosos: ( ) focais ( ) difusos / Áreas de infarto: ( ) focais ( ) difusos
Outros:

BAÇO: (X) ndn
Coloração: ( ) normocorado ( ) hipocorado ( ) congesto ( ) hemorrágico
Volume: ( ) atrofiado ( ) hipertrofiado / Consistência: ( ) firme ( ) friável
Nódulos caseosos: ( ) focais ( ) difusos / Áreas de infarto: ( ) focais ( ) difusos
Outros:

VESÍCULA BILIAR: ( ) ndn (X) cheia ( ) parede espessada ( ) cálculos ( ) parasitos
Outros:

TESTÍCULOS: ( ) ndn
Coloração: ( ) normocorado ( ) hipocorado (X) congesto ( ) hemorrágico
10 Volume: ( ) atrofiado ( ) hipertrofiado / Consistência: ( ) firme ( ) friável
Nódulos caseosos: ( ) focais ( ) difusos / Áreas de infarto: ( ) focais ( ) difusos
Outros:

OVÁRIOS: ( ) ndn
Coloração: ( ) normocorado ( ) hipocorado ( ) congesto ( ) hemorrágico
Folículos ovarianos: ( ) ausentes / No estágio: ( ) I ( ) II ( ) III ( ) IV ( ) V ( ) VI
Corpos lúteos: ( ) ausentes ( ) presentes : quantidade
Ovidutos: ( ) ndn ( ) parede espessada ( ) congesto ( ) recém-parida ( ) placas caseosas

RINS: ( ) ndn
Coloração: ( ) normocorado (X) hipocorado ( ) congesto ( ) hemorrágico
Volume: ( ) atrofiado ( ) hipertrofiado / Consistência: ( ) firme ( ) friável
Nódulos caseosos: ( ) focais ( ) difusos / Áreas de infarto: ( ) focais ( ) difusos
Outros:

ADRENAIS: (X) ndn ( ) hipocoradas ( ) congestas ( ) hemorrágicas
Outros:

GORDURA CELOMÁTICA: ( ) ndn (X) boa quantidade ( ) escassa ( ) quantidade regular ( ) excesso ( ) aavermelhada ( ) petéquias ( ) nódulos caseosos
Outros:

CELOMA: (X) ndn ( ) presença de transudato ( ) presença de exsudato ( ) parasitos

MUSCULATURA: (X) ndn

SISTEMA ÓSSEO: (X) ndn

DIAGNÓSTICO MACROSCÓPICO:

hemorragia

☐

963000000288226

FN: 2173

FICHA DE NECROPSIA

Código do animal: B. moojeni **Data de óbito:** 12/11/09 **Data de necropsia:** 17/11/09

Origem: Serp. produção **Comprimento (cm):** 90 - 10.5. **Peso (g):** 320 **Sexo:** ♂

Histórico do animal:

picado por outra moojeni

ESTADO GERAL:
(X)bom () regular () caquético

PELE: (X)ndn ( ) em ecdise ( )disecdise
() outros:

MUCOSA ORAL: ( ) ndn
Coloração: ( ) normocorada ( ) hipocorada (X) congesta ( ) cianótica ( ) hemorrágica
Estomatite: ( ) discreta ( ) moderada ( ) severa
Secreção: ( )catarral ( )caseosa Quantidade: ( ) pouca ( ) muita

TRAQUÉIA: (X) ndn
Secreção: ( ) mucosa ( ) serosa ( )purulenta ( ) caseosa (X) Sangue
Parasitos:
Outros:

PULMÃO: (X) ndn
Coloração: ( ) normocorado ( )hipocorado ( ) congesto (X) hemorrágico ( ) cianótico
Edema: ( ) discreto ( ) moderado ( ) severo
Secreção: ( ) purulenta ( ) caseosa ( ) placas caseosas ( ) nódulos caseosos
Parasitos:
Outros:

SACO AÉREO: (X) ndn
Secreção: ( ) serosa ( ) mucosa ( ) purulenta ( ) caseosa
Parasitos:
Outros:

TIREÓIDE: (X)ndn ( ) atrofiada ( ) aumentada ( ) hipocorada (X) congesta
Outros:

CORAÇÃO: ( ) ndn
Pericárdio: ( ) ndn (X)espessado ( ) hidropericárdio ( ) hemopericárdio ( ) parasitos
Miocárdio: ( ) ndn ( ) atrofiado ( ) hipertrofiado ( ) congesto ( ) hipocorado
( ) nódulos caseosos ( )áreas de infarto ( ) outros:
Valva: ( ) ndn ( )espessadas ( ) outros:
Parasitos:

11 **VASOS SANGUÍNEOS:** ( ) ndn ( ) engurgitados ( ) hipovolemia ( ) nódulos no endotélio
Outros: engurgitado na parte superior

FÍGADO: ( ) ndn
Coloração: ( ) vinhácea ( ) amarronzado ( ) amarelado ( ) congesto ( ) aparência noz moscada (X) avermelhado
Volume: ( ) atrofiado ( ) hepatomegalia / Consistência: ( ) firme ( ) friável
Nódulos caseosos: ( ) focais ( ) difusos / Áreas de infarto: ( ) focais ( ) difusos
Outros:

ESÔFAGO: (X) ndn
Mucosa: ( ) ndn ( ) congesta ( ) espessada ( ) hemorrágica ( ) ictérica
Secreção: ( ) serosa ( ) mucosa ( ) catarral ( ) purulenta ( ) caseosa ( ) enegrecida ( ) fétida
Outros:

☐

963000000291138 **FICHA DE NECROPSIA** FN: 2175

**Código do animal:** B. moojeni	**Data de óbito:** 12/11/09	**Data de necropsia:** 17/11/09	
**Origem:** Serp. modu.	**Comprimento (cm):** 82,5 - 96,5	**Peso (g):** 225	**Sexo:** ♂

Histórico do animal:

picado por outra moojeni

ESTADO GERAL:
(X) bom ( ) regular ( ) caquético

PELE: (X) ndn ( ) em ecdise ( )disecdise
( ) outros:

MUCOSA ORAL: ( ) ndn
Coloração: ( ) normocorada ( ) hipocorada (X) congesta ( ) cianótica ( ) hemorrágica
Estomatite: ( ) discreta ( ) moderada ( ) severa
Secreção: ( )catarral ( )caseosa Quantidade: ( ) pouca ( ) muita

TRAQUÉIA: (X) ndn
Secreção: ( ) mucosa ( ) serosa ( )purulenta ( ) caseosa
Parasitos:
Outros:

12 **PULMÃO:** ( ) ndn
Coloração: ( ) normocorado ( )hipocorado (X) congesto ( ) hemorrágico ( ) cianótico
Edema: ( ) discreto (X) moderado ( ) severo
Secreção: ( ) purulenta ( ) caseosa ( ) placas caseosas ( ) nódulos caseosos
Parasitos:
Outros: hemorragia na porção cranial do pulmão

SACO AÉREO: (X) ndn
Secreção: ( ) serosa ( ) mucosa ( ) purulenta ( ) caseosa
Parasitos:
Outros:

TIREÓIDE: (X) ndn ( ) atrofiada ( ) aumentada ( ) hipocorada ( ) congesta
Outros:

CORAÇÃO: (X) ndn
Pericárdio: ( ) ndn ( )espessado ( ) hidropericárdio ( ) hemopericárdio ( ) parasitos
Miocárdio: ( ) ndn ( ) atrofiado ( ) hipertrofiado ( ) congesto ( ) hipocorado
( ) nódulos caseosos ( )áreas de infarto ( ) outros:
Valva: ( ) ndn ( )espessadas ( ) outros:
Parasitos:

13 **VASOS SANGUÍNEOS:** ( ) ndn (X) engurgitados (X) hipovolemia ( ) nódulos no endotélio
→ no geral
Outros: na região da picada

FÍGADO: ( ) ndn
Coloração: ( ) vinhácea (X) amarronzado ( ) amarelado ( ) congesto ( ) aparência noz moscada
Volume: ( ) atrofiado ( ) hepatomegalia / Consistência: (X) firme ( ) friável
Nódulos caseosos: (X) focais ( ) difusos / Áreas de infarto: ( ) focais ( ) difusos
Outros:

ESÔFAGO: (X) ndn
Mucosa: ( ) ndn ( ) congesta ( ) espessada ( ) hemorrágica ( ) ictérica
Secreção: ( ) serosa ( ) mucosa ( ) catarral ( ) purulenta ( ) caseosa ( ) enegrecida ( ) fétida
Outros:

ESTÔMAGO: (X) ndn
Mucosa: ( ) ndn ( ) congesta ( ) espessada ( ) hemorrágica ( ) ictérica
Secreção: ( ) serosa ( ) mucosa ( ) catarral ( ) purulenta ( ) caseosa ( ) enegrecida ( ) fétida
Outros:

INTESTINO: (X) ndn
Mucosa: ( ) ndn ( ) congesta ( ) espessada ( ) hemorrágica ( ) ictérica
Secreção: ( ) serosa ( ) mucosa ( ) catarral ( ) purulenta ( ) caseosa ( ) enegrecida ( ) fétida
Outros:

PÂNCREAS: (X) ndn
Coloração: ( ) normocorado ( ) hipocorado ( ) congesto ( ) hemorrágico
Volume: ( ) atrofiado ( ) hipertrofiado / Consistência: ( ) firme ( ) friável
Nódulos caseosos: ( ) focais ( ) difusos / Áreas de infarto: ( ) focais ( ) difusos
Outros:

BAÇO: (X) ndn
Coloração: ( ) normocorado ( ) hipocorado ( ) congesto ( ) hemorrágico
Volume: ( ) atrofiado ( ) hipertrofiado / Consistência: ( ) firme ( ) friável
Nódulos caseosos: ( ) focais ( ) difusos / Áreas de infarto: ( ) focais ( ) difusos
Outros:

VESÍCULA BILIAR: (X) ndn ( ) cheia ( ) parede espessada ( ) cálculos ( ) parasitos
Outros:

TESTÍCULOS: ( ) ndn
Coloração: ( ) normocorado ( ) hipocorado (X) congesto ( ) hemorrágico
14 Volume: ( ) atrofiado ( ) hipertrofiado / Consistência: ( ) firme ( ) friável
Nódulos caseosos: ( ) focais ( ) difusos / Áreas de infarto: ( ) focais ( ) difusos
Outros:

OVÁRIOS: ( ) ndn
Coloração: ( ) normocorado ( ) hipocorado ( ) congesto ( ) hemorrágico
Folículos ovarianos: ( ) ausentes / No estágio: ( ) I ( ) II ( ) III ( ) IV ( ) V ( ) VI
Corpos lúteos: ( ) ausentes ( ) presentes : quantidade
Ovidutos: ( ) ndn ( ) parede espessada ( ) congesto ( ) recém-parida ( ) placas caseosas

RINS: ( ) ndn
Coloração: ( ) normocorado (X) hipocorado ( ) congesto ( ) hemorrágico
Volume: ( ) atrofiado ( ) hipertrofiado / Consistência: ( ) firme ( ) friável
Nódulos caseosos: ( ) focais ( ) difusos / Áreas de infarto: ( ) focais ( ) difusos
Outros:

ADRENAIS: ( ) ndn ( ) hipocoradas (X) congestas ( ) hemorrágicas
Outros:

GORDURA CELOMÁTICA: ( ) ndn (X) boa quantidade ( ) escassa ( ) quantidade regular ( ) excesso ( ) aavermelhada ( ) petéquias ( ) nódulos caseosos
Outros:

CELOMA: (X) ndn ( ) presença de transudato ( ) presença de exsudato ( ) parasitos

MUSCULATURA: ( ) ndn hemorragia no SC (1/3 cranial) provável local da picada.

SISTEMA ÓSSEO: ( ) ndn

DIAGNÓSTICO MACROSCÓPICO:

hemorragia

B.m

963000000299360 **FICHA DE NECROPSIA**

FN: 2170

**Código do animal:** 963000000299	**Data de óbito:** 12/11/09	**Data de necropsia:** 17/11/09
**Origem:** serp. produção	**Comprimento (cm):** 83,5 - 97,5	**Peso (g):** 250g **Sexo:** ♂

Histórico do animal: picado por outra moojeni

ESTADO GERAL:
(X) bom ( ) regular ( ) caquético

PELE: (X) ndn ( ) em ecdise ( ) disecdise
( ) outros:
na região mediana do corpo a pele está saindo

MUCOSA ORAL: (X) ndn
Coloração: ( ) normocorada ( ) hipocorada ( ) congesta ( ) cianótica ( ) hemorrágica
Estomatite: ( ) discreta ( ) moderada ( ) severa
Secreção: ( ) catarral ( ) caseosa Quantidade: ( ) pouca ( ) muita

TRAQUÉIA: (X) ndn
Secreção: ( ) mucosa ( ) serosa ( ) purulenta ( ) caseosa
Parasitos:
Outros:

PULMÃO: (X) ndn
Coloração: ( ) normocorado ( ) hipocorado ( ) congesto ( ) hemorrágico ( ) cianótico
Edema: ( ) discreto ( ) moderado ( ) severo
Secreção: ( ) purulenta ( ) caseosa ( ) placas caseosas ( ) nódulos caseosos
Parasitos:
Outros:

SACO AÉREO: (X) ndn
Secreção: ( ) serosa ( ) mucosa ( ) purulenta ( ) caseosa
Parasitos:
Outros:

TIREÓIDE: ( ) ndn ( ) atrofiada ( ) aumentada (X) hipocorada ( ) congesta
Outros:

CORAÇÃO: (X) ndn
Pericárdio: ( ) ndn ( ) espessado ( ) hidropericárdio ( ) hemopericárdio ( ) parasitos
Miocárdio: ( ) ndn ( ) atrofiado ( ) hipertrofiado ( ) congesto ( ) hipocorado
( ) nódulos caseosos ( ) áreas de infarto ( ) outros:
Valva: ( ) ndn ( ) espessadas ( ) outros:
Parasitos:

15 **VASOS SANGUÍNEOS:** ( ) ndn (X) engurgitados ( ) hipovolemia ( ) nódulos no endotélio
Outros: na região mediana do corpo (provável sítio da picada)

FÍGADO: ( ) ndn
16 Coloração: ( ) vinhácea ( ) amarronzado (X) amarelado ( ) congesto ( ) aparência noz moscada
Volume: ( ) atrofiado ( ) hepatomegalia / Consistência: (X) firme ( ) friável
Nódulos caseosos: ( ) focais ( ) difusos / Áreas de infarto: ( ) focais ( ) difusos
Outros: presença de coágulos na região caudal do fígado

ESÔFAGO: (X) ndn
Mucosa: ( ) ndn ( ) congesta ( ) espessada ( ) hemorrágica ( ) ictérica
Secreção: ( ) serosa ( ) mucosa ( ) catarral ( ) purulenta ( ) caseosa ( ) enegrecida ( ) fétida
Outros:

ESTÔMAGO: (X) ndn
Mucosa: ( ) ndn ( ) congesta ( ) espessada ( ) hemorrágica ( ) ictérica
Secreção: ( ) serosa ( ) mucosa ( ) catarral ( ) purulenta ( ) caseosa ( ) enegrecida ( ) fétida
Outros:

INTESTINO: (X) ndn
Mucosa: ( ) ndn ( ) congesta ( ) espessada ( ) hemorrágica ( ) ictérica
Secreção: ( ) serosa ( ) mucosa ( ) catarral ( ) purulenta ( ) caseosa ( ) enegrecida ( ) fétida
Outros:

PÂNCREAS: (X) ndn
Coloração: ( ) normocorado ( ) hipocòrado ( ) congesto ( ) hemorrágico
Volume: ( ) atrofiado ( ) hipertrofiado / Consistência: ( ) firme ( ) friável
Nódulos caseosos: ( ) focais ( ) difusos / Áreas de infarto: ( ) focais ( ) difusos
Outros:

BAÇO: ( ) ndn
Coloração: ( ) normocorado ( ) hipocorado (X) congesto ( ) hemorrágico
Volume: ( ) atrofiado ( ) hipertrofiado / Consistência: ( ) firme ( ) friável
Nódulos caseosos: ( ) focais ( ) difusos / Áreas de infarto: ( ) focais ( ) difusos
Outros:
autólise

VESÍCULA BILIAR: (X) ndn ( ) cheia ( ) parede espessada ( ) cálculos ( ) parasitos
Outros:

TESTÍCULOS: ( ) ndn
Coloração: ( ) normocorado ( ) hipocorado (X) congesto ( ) hemorrágico
17 Volume: ( ) atrofiado ( ) hipertrofiado / Consistência: ( ) firme ( ) friável
Nódulos caseosos: ( ) focais ( ) difusos / Áreas de infarto: ( ) focais ( ) difusos
Outros:

OVÁRIOS: ( ) ndn
Coloração: ( ) normocorado ( ) hipocorado ( ) congesto ( ) hemorrágico
Folículos ovarianos: ( ) ausentes / No estágio: ( ) I ( ) II ( ) III ( ) IV ( ) V ( ) VI
Corpos lúteos: ( ) ausentes ( ) presentes : quantidade
Ovidutos: ( ) ndn ( ) parede espessada ( ) congesto ( ) recém-parida ( ) placas caseosas

RINS: (X) ndn
Coloração: ( ) normocorado ( ) hipocorado ( ) congesto ( ) hemorrágico
Volume: ( ) atrofiado ( ) hipertrofiado / Consistência: ( ) firme ( ) friável
Nódulos caseosos: ( ) focais ( ) difusos / Áreas de infarto: ( ) focais ( ) difusos
Outros:

ADRENAIS: ( ) ndn ( ) hipocoradas (X) congestas ( ) hemorrágicas
Outros:

GORDURA CELOMÁTICA: ( ) ndn (X) boa quantidade ( ) escassa ( ) quantidade regular ( ) excesso ( ) aavermelhada ( ) petéquias ( ) nódulos caseosos
Outros:

CELOMA: (X) ndn ( ) presença de transudato ( ) presença de exsudato ( ) parasitos

MUSCULATURA: ( ) ndn avermelhada na área da picada

SISTEMA ÓSSEO: (X) ndn

DIAGNÓSTICO MACROSCÓPICO:
hemorragia interna
